# Nasal Reconstruction in Granulomatosis with Polyangiitis: A Two Decade Review

**DOI:** 10.1089/fpsam.2021.0348

**Published:** 2023-01-18

**Authors:** Andres Gantous, Rodrigo Fortunato Fernández-Pellón Garcia

**Affiliations:** Division of Facial Plastic and Reconstructive Surgery, Department of Otolaryngology-Head & Neck Surgery, Temerty Faculty of Medicine, University of Toronto, Toronto, Canada.

## Abstract

**Background:**

Granulomatosis with polyangiitis (GPA) leads to progressive destruction of the nasal tissues resulting varying degrees of saddle deformity and nasal obstruction. Reconstructive techniques are numerous, but there are no large series reporting their results.

**Objective:**

This study sought to measure complications and outcomes after rhinoplasty for GPA.

**Methods:**

We conducted a retrospective review of 42 patients with GPA who underwent nasal reconstruction of saddle nose deformity between 2005 and 2019 using primarily costal cartilage and soft tissue grafts.

**Results:**

Thirty-six patients met the criteria for inclusion. All were followed for a minimum of 12 months. Six patients required revision surgery due to infection or GPA flare ups. Five patients had complications. All patients were given a questionnaire at 12 months to rate their degree of satisfaction with their appearance and breathing.

**Conclusion:**

The findings of this study suggest that the use of strong cartilage grafts and the timing of surgery result in improvement in breathing and appearance after rhinoplasty in patients with GPA.

Clinical Trial Registration number: REB # 21-125.

**KEY POINTS**

**Question:** When is it prudent to attempt to repair a saddle nose deformity in patients with an autoimmune disorder and what type of operation should be chosen to improve both how they look and breathe?

**Findings:** Patients who underwent rhinoplasty surgery to reconstruct the damage done on the nose by an autoimmune disorder showed that the appropriate choice of surgery can result in a predictable and sustained improvement.

**Meaning:** Patients and their surgeon in this study reported satisfaction after rhinoplasty for an autoimmune disorder than can erode the nasal cartilages and deform the nose.

## Introduction

Saddle nose deformities can be devastating and, in the presence of a disease such as granulomatosis with polyangiitis (GPA), they add insult to injury to a disease that often affects multiple organ systems. These patients are subject to a high level of psychological and emotional distress and their quality of life is greatly diminished.^1^ As such, patients are often eager to accept even the most minimal of improvement in their condition.^2^ A multidisciplinary approach that includes the rheumatologist, sinus surgeon, and an experienced rhinoplasty surgeon is required to achieve the best possible outcome.^3^

The nasal deformity caused by GPA is due to a progressive loss of structural support of the nose. The vasculitis and granulomatous inflammatory changes in the nasal lining leads to a loss of septal cartilage and, eventually, changes in the nasal and facial bones.^4,5^ This leads to a collapse of the nasal framework, leading to varying degrees of saddle deformity, valvular obstruction, nasal foreshortening, and injury to the skin-soft tissue envelope (S-STE) of the nose.^6,7^ Most patients will also suffer from crusting, bleeding, and chronic and recurrent rhinosinusitis.^8^

The degree of the deformity and loss of structural support, the presence or absence of a septal perforation, as well as the integrity of the S-STE of the nose will dictate the type of grafts and grafting materials.^9,10^ Costal cartilage has proven to be reliable, readily available, and easy to use.^11^ There are many issues that can occur with the use of costal cartilage, and these have been abundantly discussed in other publications.^12^

Vertical support of the lower two thirds of the nose is achieved using strong columellar struts, septal extension grafts (SEG), or septal replacement grafts (SRG).^13–15^ We have found that these grafts need to be slightly larger and thicker than when used in non-GPA cases. They are, most often, attached to the anterior nasal spine through periosteal sutures or bone tunnels.^16^ Longitudinal support is always required, and the choice of grafting will also depend on the degree of nasal collapse. Mild to moderate saddle deformities (type I and II)^17^ can be dealt with the use of extended spreader grafts secured to the columellar strut, SEG, or SRG. The extended spreaders can often be designed to provide some vertical height as well.^18^

The use of a single extended spreader graft is at times enough, particularly if contour grafting is planned.^15^ The more severe deformities (type III–V) are dealt with solid dorsal grafting. Cantilever dorsal grafts, a workhorse of nasal reconstruction, have been used earlier in this series.^2^ L-shaped grafts provide the strongest form of reconstruction, and these can be shaped from a single piece of rib cartilage or put together in situ.^19^ The use of a tongue in groove attachment between the caudal aspect of the dorsal segment and the vertical support graft has been the preferred method for such reconstruction for the past 10 years (Supplementary Fig. S1a).^17,20^ Perichondrium grafts are sutured on the ventral side of the cephalic aspect of the dorsal graft to allow for better adhesion to the gently rasped underlying nasal bones.

Perichondrium and/or fascia are also used as an onlay graft over the dorsal graft to better camouflage the solid cartilage graft.^21^ Contour grafting overlying the longitudinal dorsal grafts is sometimes required. We have used morselized cartilage,^22^ cartilage gel, finely diced cartilage covered with fascia,^23^ and, more recently, diced cartilage glue grafts.^24^ We have had good results with all these grafts, but these “finer” grafting choices are more susceptible to resorption in the event of a flare up. Lateral wall support is also often necessary, and this is provided by using lateral crural underlay grafts,^25^ batten grafts,^26,27^ and articulated rim grafts.^28^ Lateral wall grafts carved out of thin sheets of costal cartilage have been used in severe deformities with good results. The type of graft used is individualized to the patient's needs.

The early treatment of these deformities is ideal when possible. The mild to moderate saddle deformity (type I and II) with minimal or no foreshortening of the nose will result in a better cosmetic and functional result than that attainable in more advanced cases.^29^ The timing for surgery is of paramount importance. In general, patients undergoing surgery must be in a sustained remission for 6 months or more or on a stable low-dose maintenance immunosuppressive regimen. If systemic treatment has already been stopped, we wait 6 months before considering surgery. The ongoing treatment must be maintained.^30^ The basic biologic markers (C-reactive protein, creatinine, and urinalysis) as well as the antineutrophil cytoplasmic antibodies (cANCA) need to be checked, cANCA+patients tend to relapse more often, and these patients need to be informed and made aware of this.^31^

The purpose of this study is to analyze our results among patients undergoing rhinoplasty to treat the effects of GPA.

## Materials and Methods

This is a retrospective cohort study conducted with patient data from May 2005 to January 2019. Permission for this study was granted by the Ethics board of Toronto Academic Health Sciences Network (TAHSN). Only patients with proven GPA presenting with nasal saddle deformity and various degrees of nasal foreshortening, collapse, and airway obstruction (type I–IV) were included in this review. All the operations were performed by the principal author at a university teaching hospital.

All patients were managed by a rheumatologist and the timing of their surgery was carefully managed in accordance with their recommendations. All underwent nasal reconstruction through an open rhinoplasty approach with costal cartilage as the primary grafting material. Costal cartilage was harvested through an infra-mammary incision, using an inferior strip preservation technique.^32^

The grafts that were more commonly used were cantilever dorsal grafts,^33^ L-grafts,^19^ tongue-in- groove grafts,^17,20^ columellar struts,^13^ SRG^14^ and SEG,^13,15,34^ spreader^35^ and extended spreader grafts,^15^ batten^26^ and lateral crural underlay grafts,^25^ plumping grafts,^36^ fascia and perichondrium onlay^37^ and underlay grafts. The dorsal grafts were fixated in a variety of ways, although some required no fixation due to the size of the dorsal pocket. Transcutaneous K-wire fixation, transcutaneous sutures, internal sutures to the upper lateral cartilages, and TISSEEL™ were used. Most dorsal grafts had perichondrium sutured to the ventral surface of the graft.

Data were collected on patient demographics, preoperative structural defects, specific types of grafts used for reconstruction, fixation materials, and surgical outcome.

A successful surgical outcome was considered when there was clinical evidence of improvement of the preoperative deformity and there was no need for a revision surgery. Most importantly though, success was measured by patient satisfaction. If patients felt that the treatment provided was beneficial, we considered the result as satisfactory. We realize that there is a lack of objectivity in this. We did not start using validated nasal outcomes scores until the later part of the timeline of this study so our information in that regard is incomplete.^38^

A short questionnaire was given to 36 patients at a minimum of 12 months postoperative to assess the improvement in breathing quality and satisfaction with their appearance. This questionnaire consisted of two questions with three responses each. Question 1 addressed the subjective improvement in breathing. The patients were asked to rate their breathing quality as worse, same, or improved. Question 2 asked patients how satisfied they were with the cosmetic appearance of their nose, which they rated as unsatisfied, satisfied, or very satisfied.

## Results

Forty-two patients with GPA underwent nasal reconstruction during this time. Thirty-six patients met the inclusion criteria. Seven patients were male and 29 were female. The age range was from 17 to 68 years. All patients were followed for a minimum of 1 year. The average follow-up was 27 months (Tables 1 and 2). All patients had varying degrees of nasal collapse with saddle deformity and nasal foreshortening. Seven patients (19.4%) had large septal perforations at the time of surgery. Seven patients had an attempt at nasal reconstruction done elsewhere: one patient had solid silicone implants, one had Goretex™ and five had conchal cartilage grafts.

**Table 1.** Summary of patient demographics, grafts used, fixation method, status of septum, prior surgery, revision surgery, complications, and follow-up

Age	Gender	Longitudinal graft	Vertical graft	Soft tissue/contour graft	Graft fixation	Septal perforation	Prior surgery	Complications	Revision surgery	Follow-up
40	F	Cantilever	SRG	Contour	Tiseel	N		Dorsal infection	1	139
34	M	L-graft	SRG	Perichondrium	K-wire	N	Y			12
48	F	Cantilever	SRG		Suture	N				40
27	F	Cantilever	Strut	NL angle plumping grafts	Suture	Y		Columellar necrosis	1	128
35	F	L-graft	Strut	Contour	Suture	N				12
37	F	L-graft	SEG	Perichondrium	Suture	N				12
49	F	Cantilever	Strut		TC suture	Y			1	24
33	F	Cantilever	Strut/septal grafts			Y				12
31	F	L-graft	SRG	Perichondrium	Suture	N				13
17	M	L-graft	SRG/spreaders	F/P/contour	Suture	N				12
46	F	Cantilever	SRG	Perichondrium	Suture	N				16
28	M	L-graft	Strut	Perichondrium		N				12
68	F	L-graft	SRG	F/P	K-wire	N				26
27	M	L-graft	SRG/spreaders/LCUS	P/contour		N		Nasal infection	1	24
69	M	L-graft	SRG	P/contour	K-wire	N				18
31	F	L-graft	SRG	P/contour		N				14
56	F	L-graft	Strut		TC suture	N	Y			12
40	F	L-graft	SRG	Perichondrium		N			1	25
19	F	Cantilever	Strut	Contour		N	Y			12
33	F	Cantilever	Strut		Tiseel	N	Y			12
47	F	L-graft	SEG		Suture	N				13
37	F	L-graft	SRG	P/contour		Y				13
55	F	Cantilever	SRG	Contour	TC suture	N	Y	Columellar infection		22
32	M	L-graft	SRG/LCUS	Perichondrium	Suture	N				20
26	F	Cantilever	Strut		Suture	Y				16
39	F	L-graft	SEG	Perichondrium	Suture	N				18
52	F	L-graft	SRG/spreaders	F/P	Suture	N				12
46	F	L-graft	SRG	F/P/contour		N	Y			15
31	F	L-graft	SRG	Perichondrium	K-wire/suture	N				12
20	F	L-graft	SRG/spreaders	F/P/contour	K-wire/suture	N				12
35	M	L-graft	SRG	F/P/contour		N	Y			32
24	F		Strut/spreaders	Contour	Suture	Y		Donor site infection	3	118
42	F	Cantilever/lateral dorsal grafts		P/contour	Suture	N				32
38	F	Cantilever	SRG	Perichondrium	Suture	N				21
46	F		SRG/spreaders	Contour	Suture	Y				22
44	F	L-graft	SRG		Suture	N				24

F, female; LCUS, lateral crural underlay strut; M, male; SEG, septal extension grafts; SRG, septal replacement grafts.

**Table 2.** Univariate analysis of age, gender, and grafts used in patients with and without complications

	All cases	Complications	No complications	*p* ^*^
Patients	36	5 (13.8%)	31 (86.11%)	<0.05
Gender				
Male	29 (80.6 (%)	4 (13.79%)	25 (86.20%)	>0.05
Female	7 (19.4%)	1 (14.28%)	6 (85.71%)	
Age				
Mean	38.38 ± 12.30	34.60 ± 12.97	39 ± 12.3	>0.05
Range	17–69	24–55	17–69	
Median	37	17	27	
L-graft	21	1	20	>0.05
w/o L-graft	15	4	11	
Strut	9	2	7	>0.05
w/o strut	27	3	24	
SRG	26	3	23	>0.05
w/o SRG	10	2	8	
Spreaders	10	3	7	>0.05
w/o spreaders	26	2	24	
Fascia	8	0	8	>0.05
w/o fascia	28	5	23	
Perichondrium	21	1	20	>0.05
w/o perichondrium	15	4	11	
Contour graft	14	4	10	>0.05
w/o contour graft	22	1	21	
K-wire	7	0	7	>0.05
w/o K-wire	29	5	24	

^*^ *p*-Values were calculated using a chi square test or Fisher's exact test for discrete variables and a *t*-test for continuous variables.

Twenty-three patients (63.8%) had SRG, 3 patients (8.33%) had SEG, 9 patients (25%) had columellar struts, and 1 patient (2.77%) did not have any vertical support grafting. Twelve patients (33.3%) had a dorsal cantilever graft, 21 patients (58.33%) had an articulated L-graft, and 3 patients (8.33%) had extended spreader grafts as the sole form of longitudinal support. Two patients (5.55%) had unilateral spreader grafts and one patient (2.77%) had bilateral spreader grafts placed under the L-graft. Fourteen patients (38.8%) had contour grafting over the main longitudinal graft.

Twenty-one patients (58.33%) had perichondral grafts, 6 patients (16.66%) had fascial grafts. One patient (2.77%) had a septal reconstruction with two cartilage grafts, two patients (5.55%) had lateral crural underlay grafts, one patient (2.77%) had columellar and nasolabial plumping grafts, and one patient (2.77%) had bilateral lateral dorsal grafts. The dorsal grafts were fixed with K-wires in a transcutaneous manner in 7 patients (19.44%), with transcutaneous sutures in 4 patients (11.11%), with internal sutures in 16 patients (44.44%), and with TISSEEL in 2 patients (5.55%). The rest had no other form of fixation.

Seven patients (19.44%) required a revision operation (one patient had three revision operations). One patient needed a revision for a postoperative infection that eroded the grafts. One patient required a melolabial flap to reconstruct the columella. All the other revisions were necessary due to nasofacial changes secondary to progression of the disease.

**Fig. 1.**
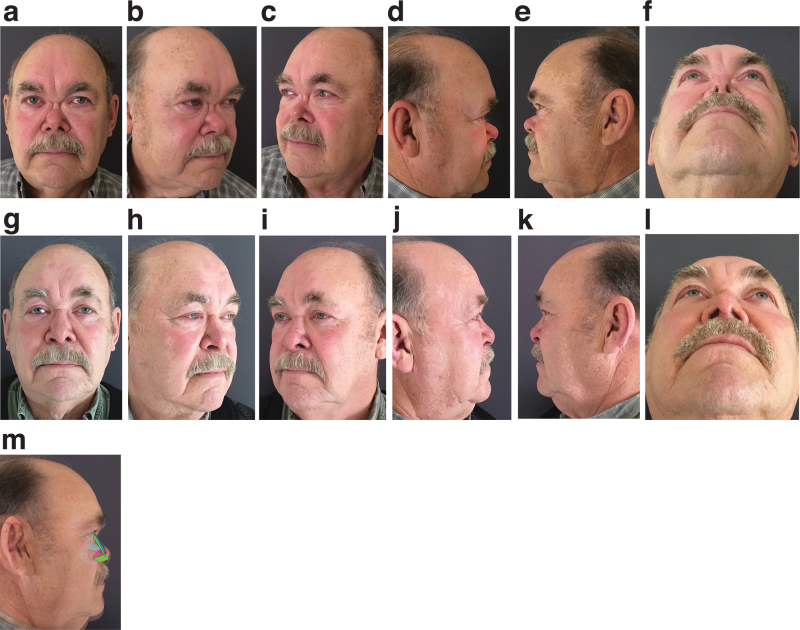
Patient 1: Moderate saddle deformity with nasal foreshortening. Preoperative: **(a)** frontal; **(b)** oblique right; **(c)** oblique left; **(d)** lateral right; **(e)** lateral left; **(f)** basal, and 1-year postoperative **(g)** frontal; **(h)** oblique right; **(i)** oblique left; **(j)** lateral right; **(k)** lateral left; **(l)** basal; **(m)** reconstructed with a septal replacement graft, extended spreader grafts, onlay solid cartilage graft over mid third, gently morselized cartilage onlay graft over superior third, rectus fascia onlay graft. Patient 2: Moderate to severe saddle deformity with significant foreshortening. Preoperative and 1 year postoperative. Preoperative: **(a)** frontal; **(b)** oblique right; **(c)** oblique left; **(d)** lateral right; **(e)** lateral left; **(f)** basal, and 1-year postoperative **(g)** frontal; **(h)** oblique right; **(i)** oblique left; **(j)** lateral right; **(k)** lateral left; **(l)** basal; **(m)** reconstructed with a septal replacement graft, extended spreader grafts with vertical positioning, onlay solid cartilage graft spanning the upper and middle thirds and rectus fascia onlay graft. Patient 3: Severe saddle deformity and nasal collapse. Preoperative: **(a)** frontal; **(b)** oblique right; **(c)** oblique left; **(d)** lateral right; **(e)** lateral left; **(f)** basal, and 1-year postoperative **(g)** frontal; **(h)** oblique right; **(i)** oblique left; **(j)** lateral right; **(k)** lateral left; **(l)** basal; **(m)** reconstructed with a septal replacement graft, tongue-in-groove dorsal graft, second solid cartilage onlay graft spanning the upper and middle thirds and rectus fascia onlay graft.

Three patient example cases are shown to illustrate the range of deformities (Fig. 1, Supplementary Figs. S2 and S3).

One patient had a serious postoperative complication developing a columellar necrosis 2 weeks postoperative, one patient developed a postoperative infection that was ignored, and the grafts resorbed, one patient had a donor site infection (inframammary), one (2.77%) patient had a dorsal infection that resolved with antibiotics, and one (2.77%) patient had a columellar infection that also resolved with oral antibiotics.

Thirty-six patients were given a short questionnaire at a minimum of 12 months postoperative to assess qualitatively the improvement in breathing quality and the cosmetic satisfaction (Supplementary Figs. S4 and S5).

## Discussion

This study reviews the results of a large series of patients suffering from GPA who underwent rhinoplasty to improve both the functional and cosmetic sequelae of the disease. No differences were found in terms of age, gender, and type of grafts used in those who developed complications. The satisfaction scores for cosmetic appearance reported 47.2% as very satisfied, 44.4% as satisfied, and 8.3% as unsatisfied. The functional satisfaction scores reported 77.7% improvement in breathing and 22.3% with no change.

The surgical results obtained in this study are comparable with those reported in other series.^2,3,7,10,19,29,39^ Rhinoplasty in the presence of an autoimmune disorder such as GPA can be successful in restoring structure and cosmesis to the nose as well as in improving breathing. The operation is fraught with an increased complication rate due to the impaired blood supply caused by the vasculitis, the hypercoagulable state that is inherent in GPA and the immune modulating medications that patients are usually on.^2,5,7,8,31^

Patients undergoing surgery must be in a sustained remission for 6 months or more or on a stable low-dose maintenance immunosuppressive regimen. If systemic treatment has already been stopped, we wait 6 months before considering surgery. Some patients will have recurrence of their disease, and this can lead to graft resorption in some cases. It has been our observation that the use of thicker and sturdier cartilage grafts when restructuring the longitudinal and vertical support seem to withstand disease relapses better.

Septal perforation repair was not attempted in any of the seven patients in this series where it was present. Although normal laminar air flow cannot be restored without a septal perforation repair, it is our observation that restoring normal structure and shape to the nose results in significant subjective airway improvement.^40,41^

Our study has several important limitations, most importantly because this database was created with retrospective information from our patient charts. Which brings into play limitations in terms of accuracy of the recorded observations and the transcription records that can introduce errors despite all our efforts. We have not addressed the reconstruction of the inner lining of the nose and never were able to consider free tissue transfer for the internal reconstruction of the nasal cavity.^42^ We are unable to conclude that a multidisciplinary approach in the management of these patients results in better outcomes because we do not have a control group to compare with. Nonetheless, we feel comfortable that we have been able to gather and present important data that should help other surgeons manage these complex cases.

GPA is a rare disease, and, as such, prospective studies would be hard to do. Introducing proper validated outcome measures questionnaires for future evaluations is essential.

## Conclusion

Rhinoplasty in patients suffering from GPA can be safe and reliable operation. The findings of this study suggest that the use of strong cartilage grafts and the timing of surgery result in improvement in breathing and appearance after rhinoplasty in patients with GPA.

## Supplementary Material

Supplemental data

Supplemental data

Supplemental data

Supplemental data

Supplemental data
