# Supplementary figures and images for: Nasal Reconstruction in Granulomatosis with Polyangiitis: A Two Decade Review
Source: Facial Plast Surg Aesthet Med. 2023 Jan 18;25(1):61–7. doi: 10.1089/fpsam.2021.0348 (PMC9885542; doi:10.1089/fpsam.2021.0348)

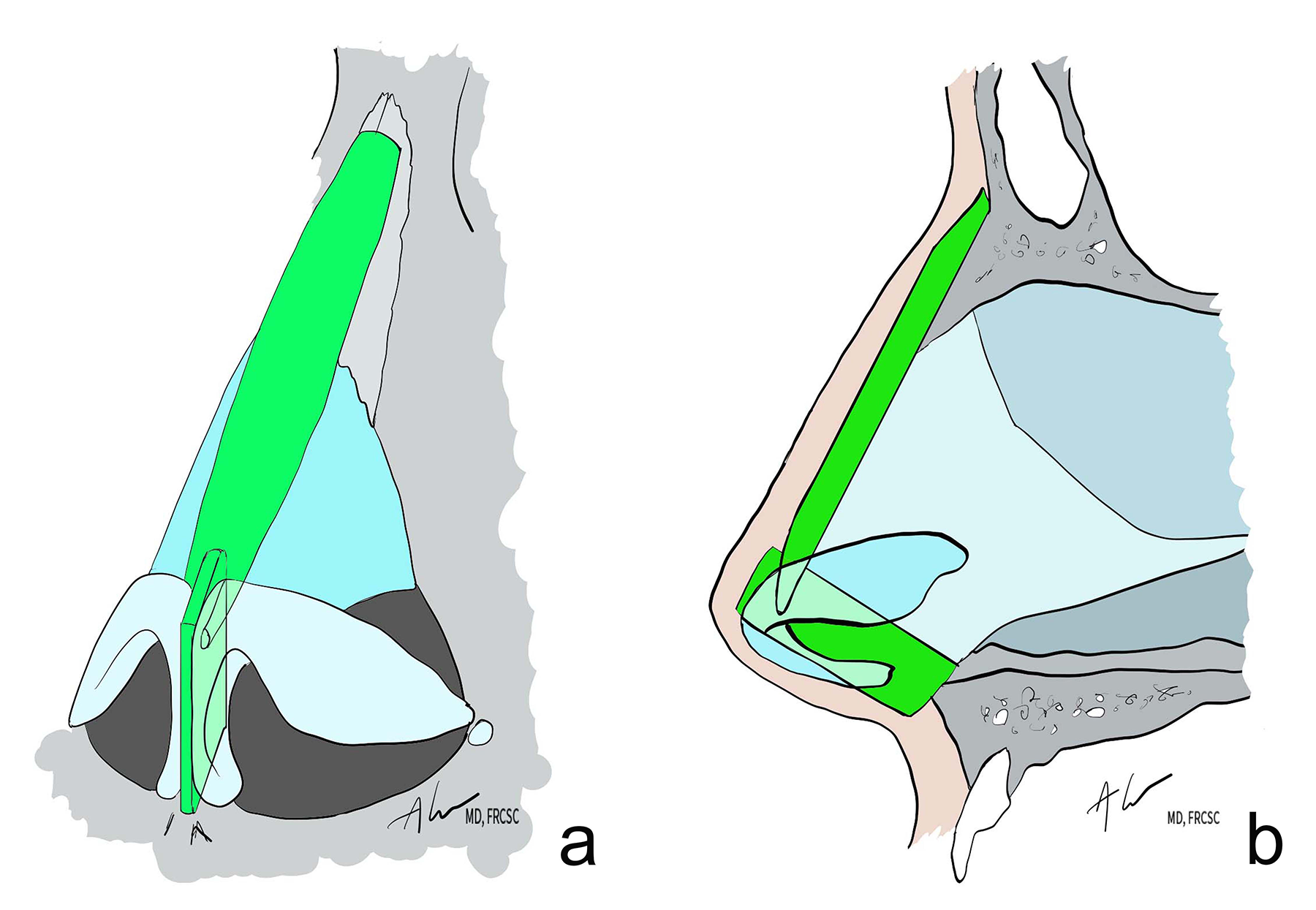

Supplement: Supplemental data [file Suppl_FigS1.docx]

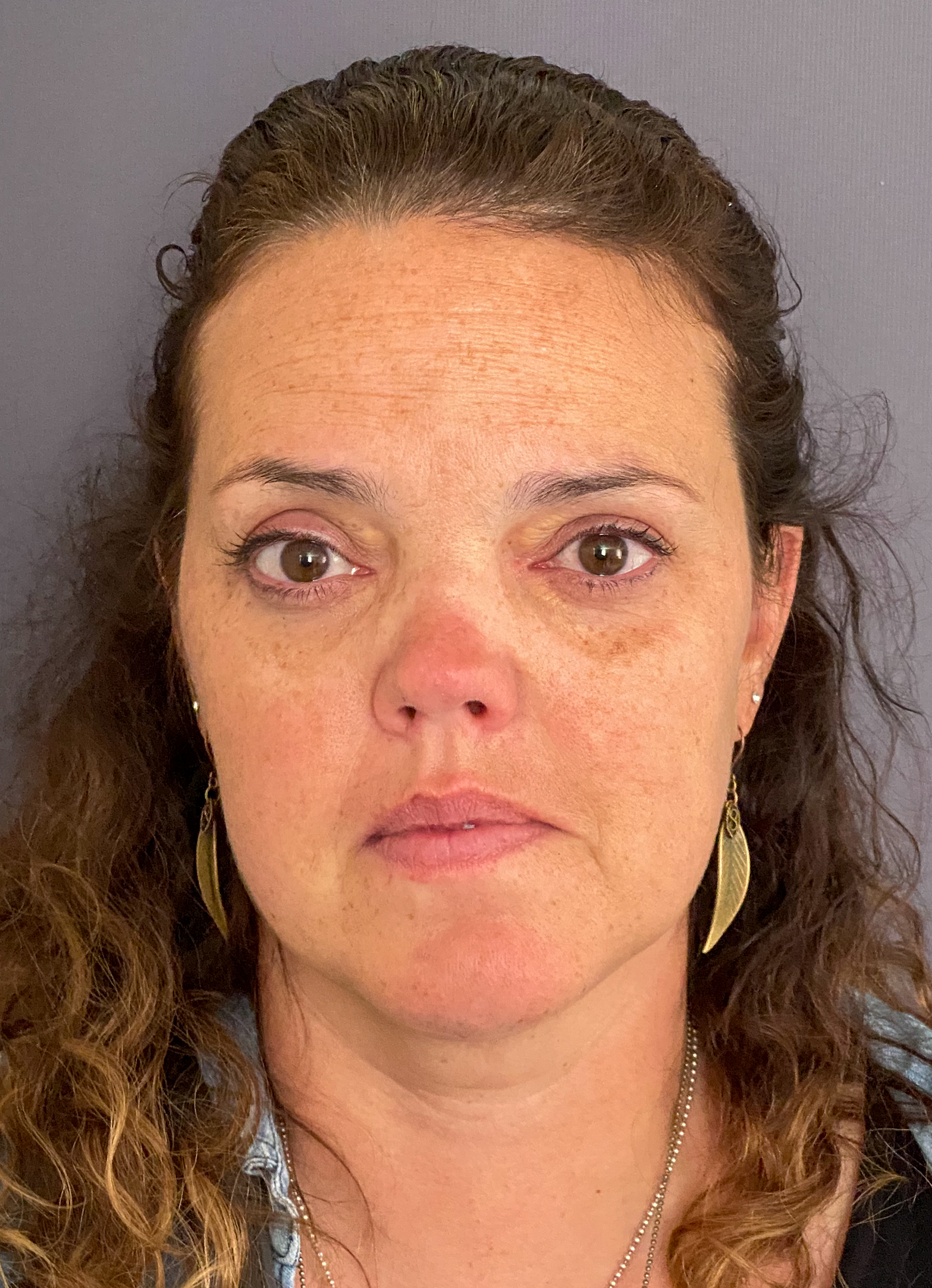

Supplement: Supplemental data [file Suppl_FigS2.zip › SuppFig2a.tif]

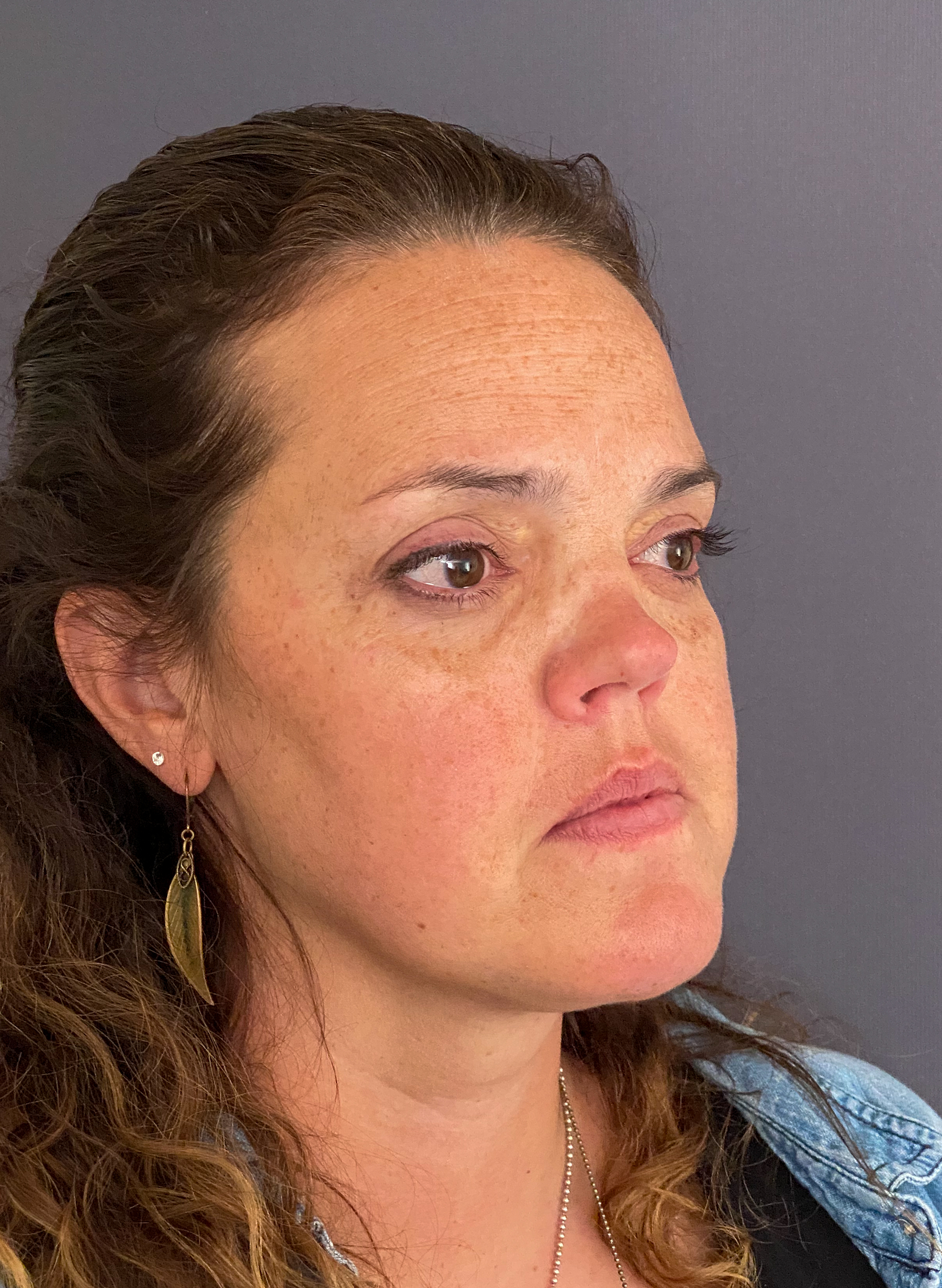

Supplement: Supplemental data [file Suppl_FigS2.zip › SuppFig2b.tif]

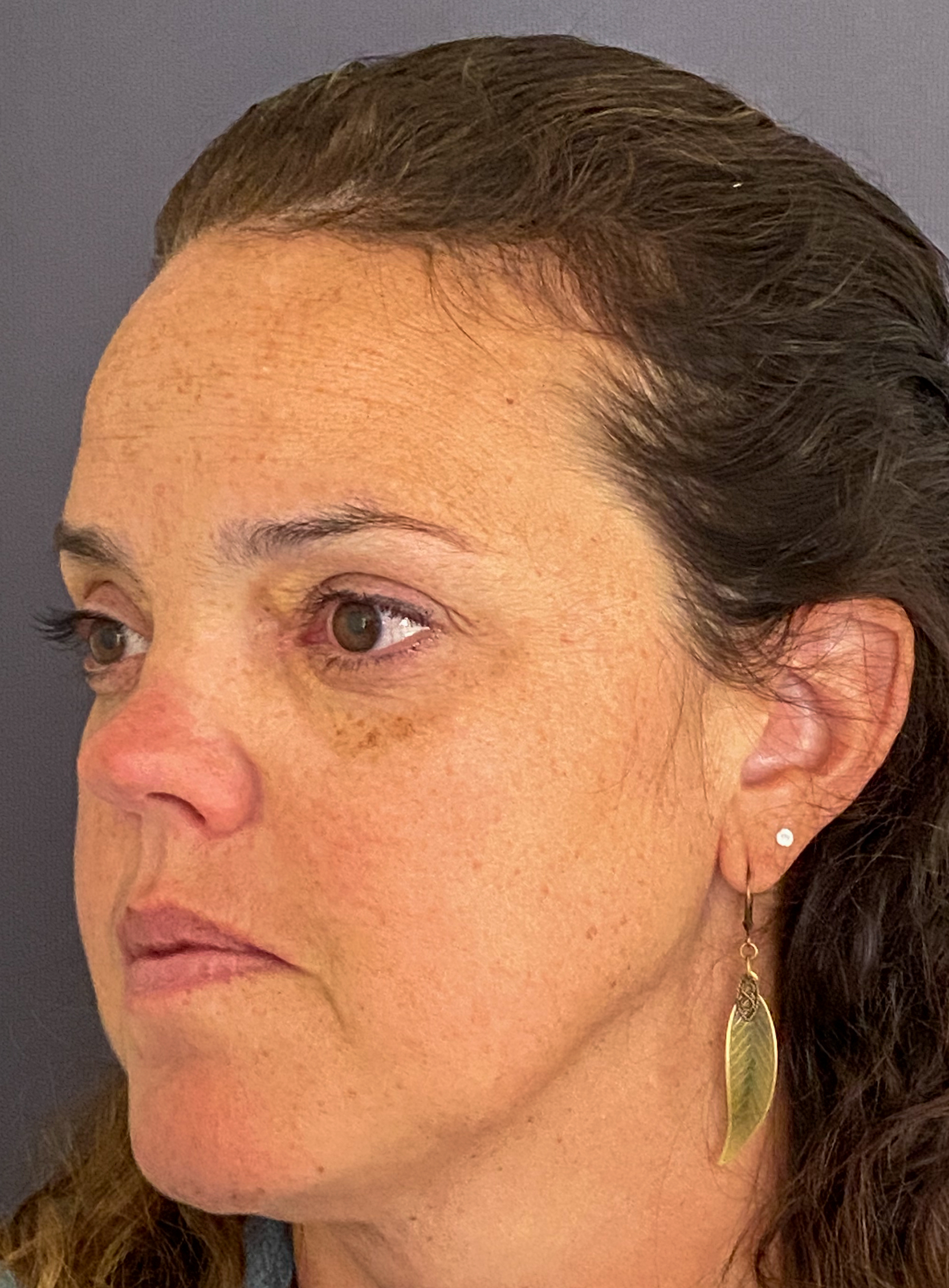

Supplement: Supplemental data [file Suppl_FigS2.zip › SuppFig2c.tif]

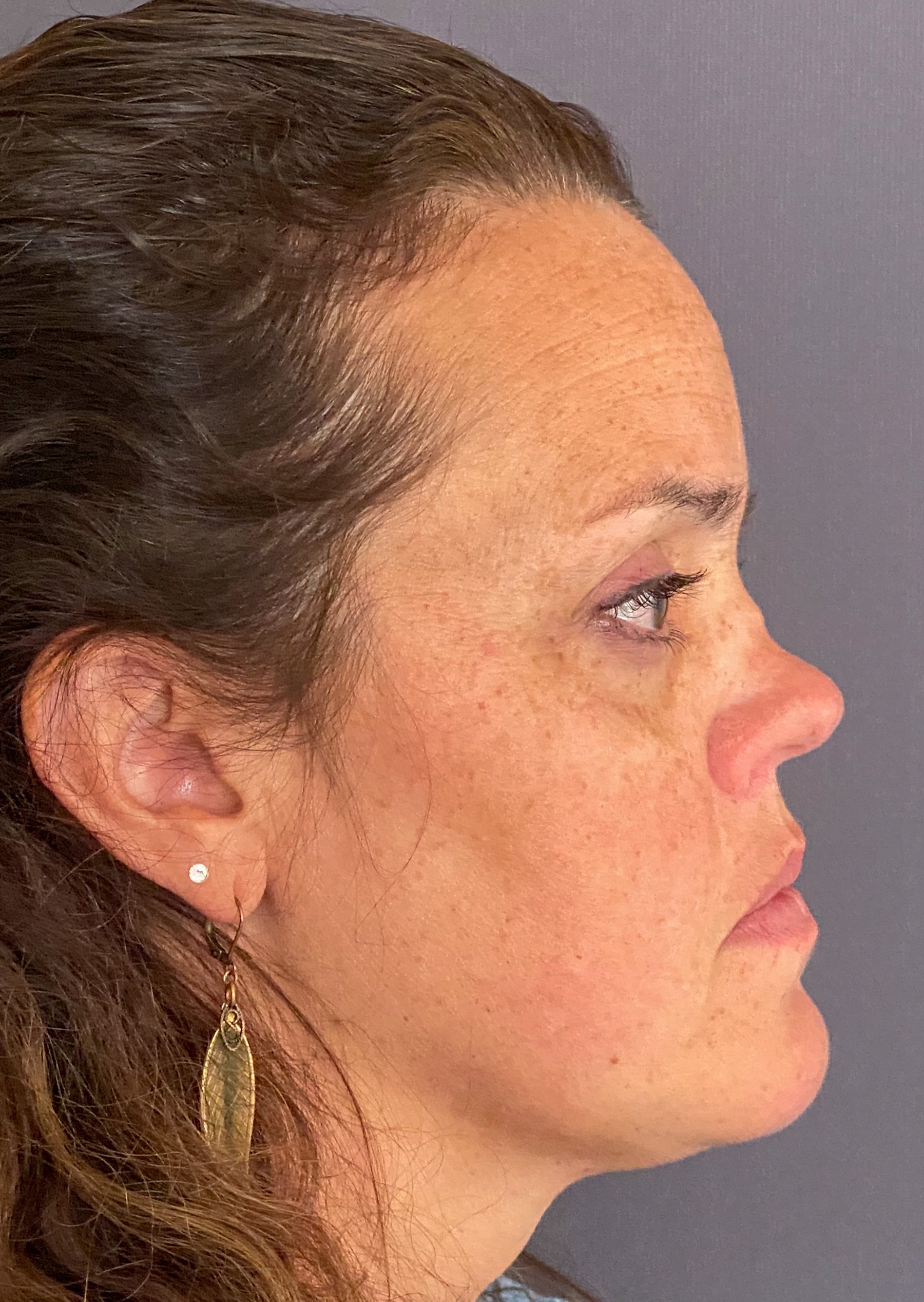

Supplement: Supplemental data [file Suppl_FigS2.zip › SuppFigure2d.tif]

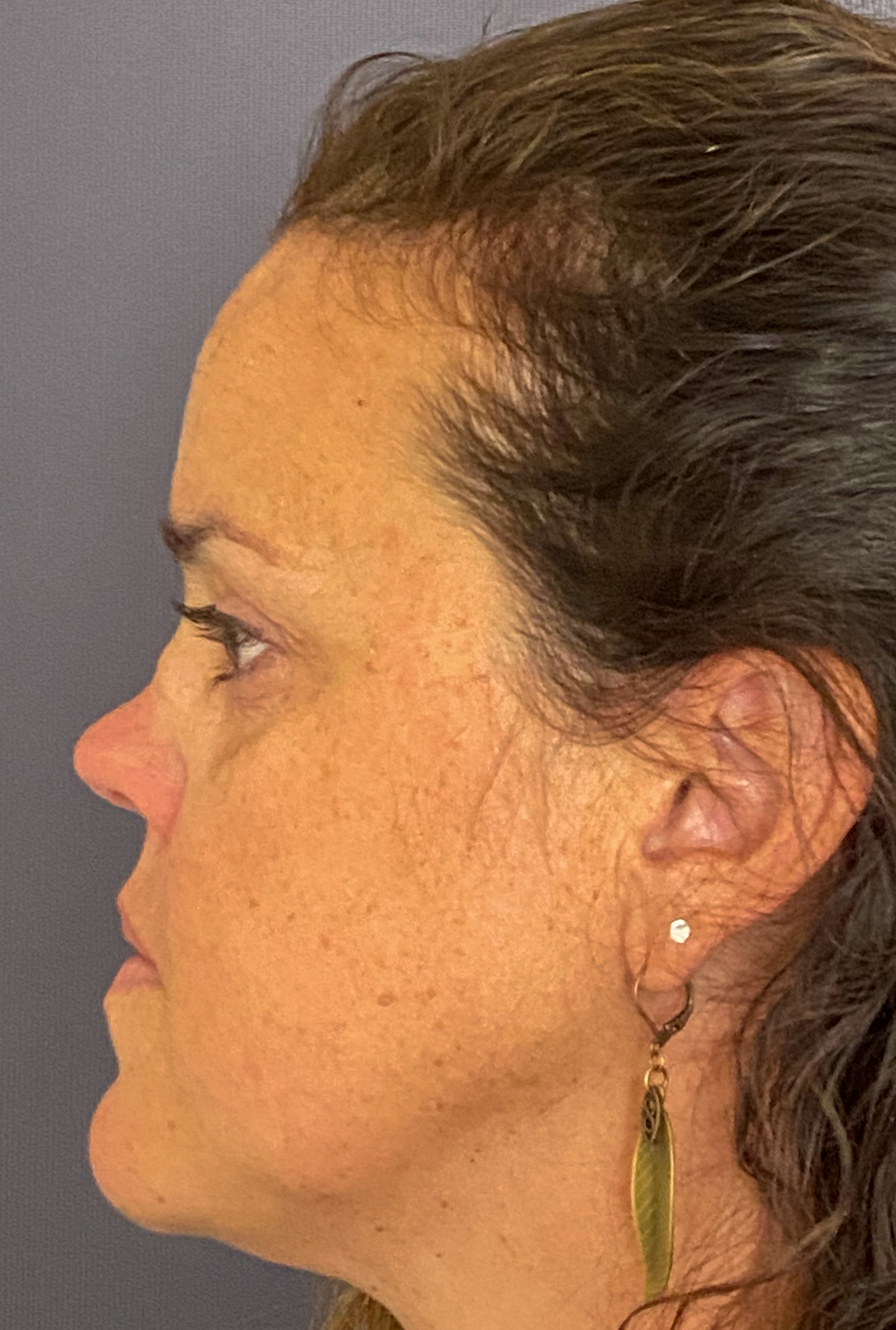

Supplement: Supplemental data [file Suppl_FigS2.zip › SuppFigure2e.tif]

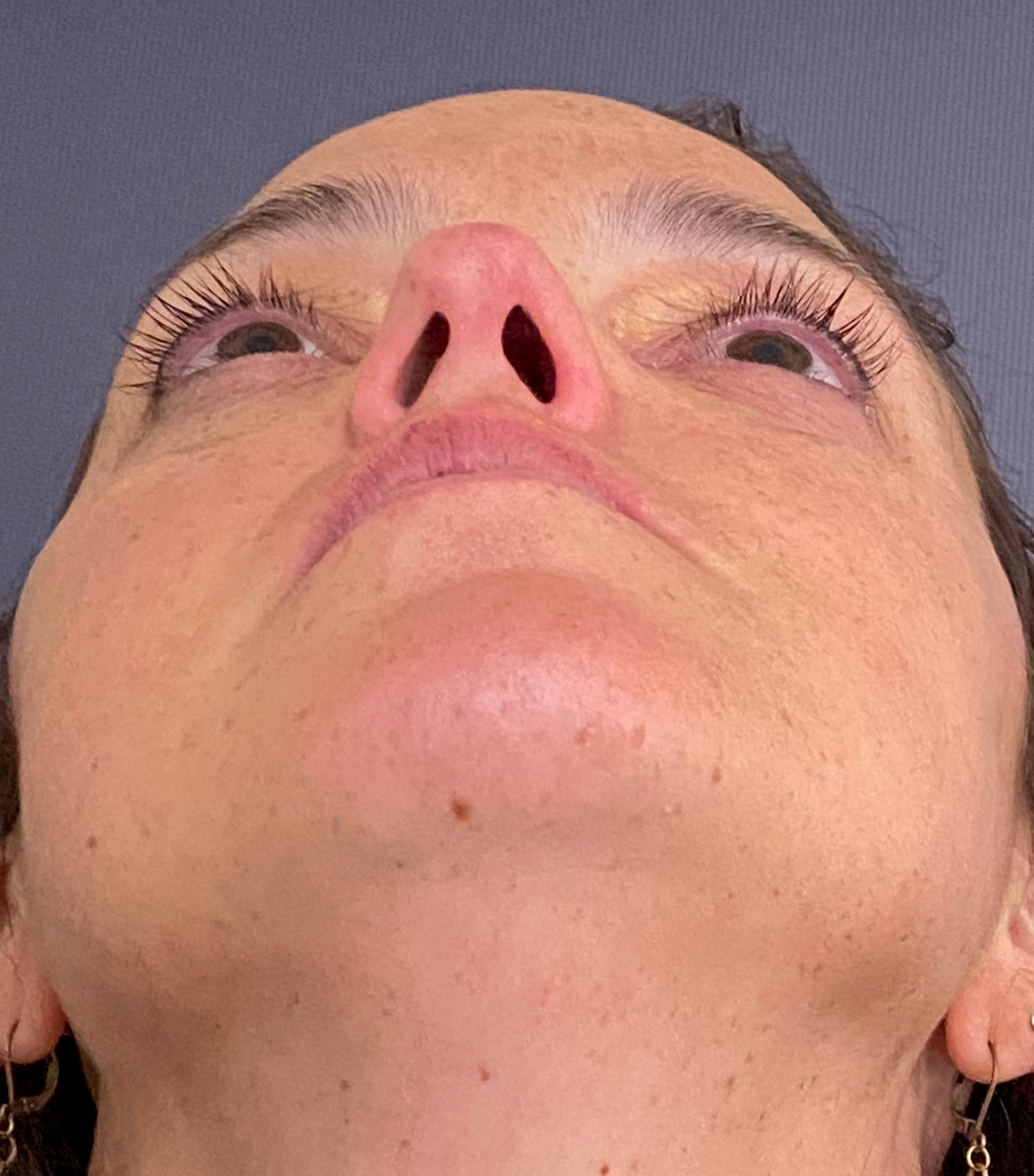

Supplement: Supplemental data [file Suppl_FigS2.zip › SuppFigure2f.tif]

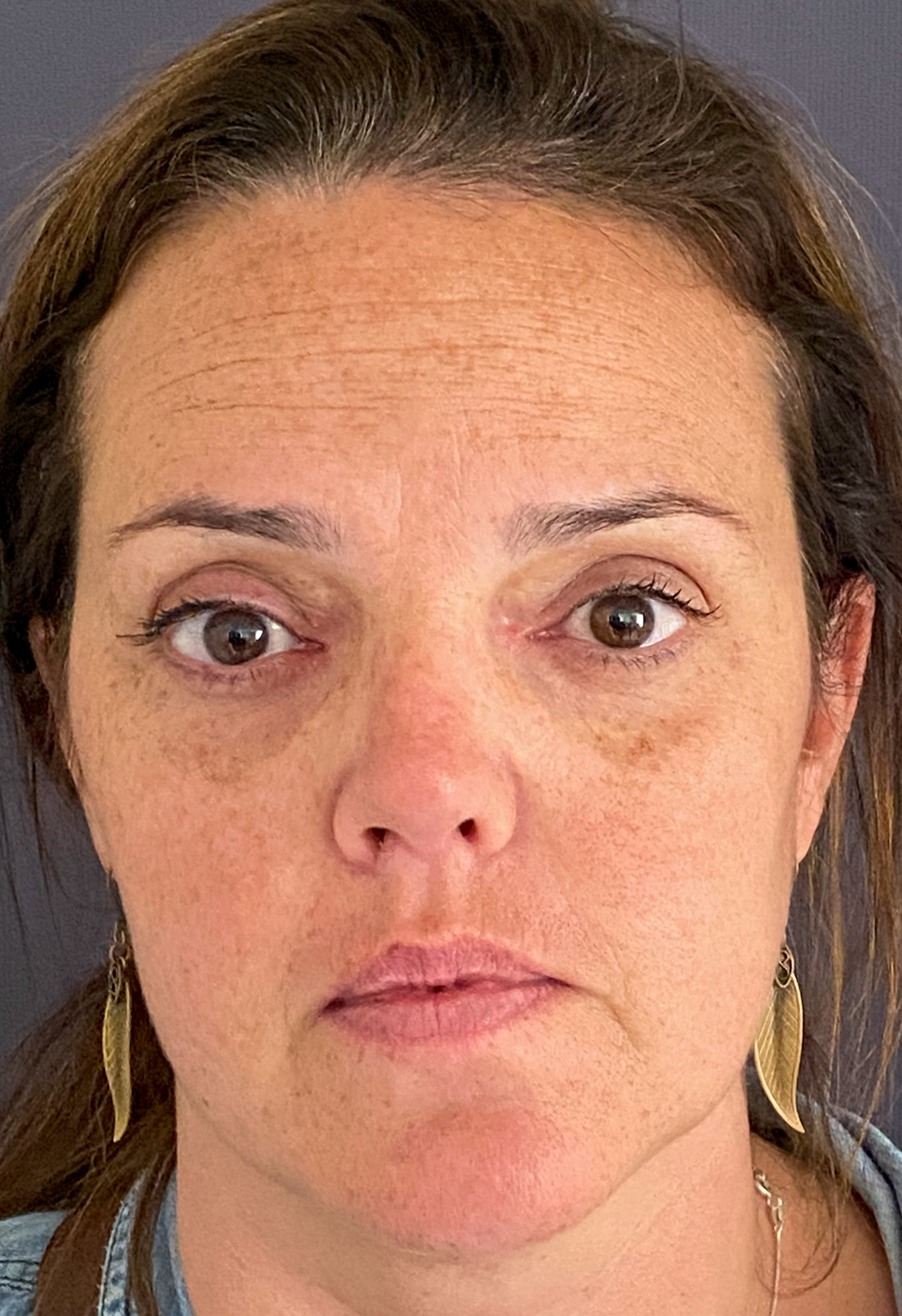

Supplement: Supplemental data [file Suppl_FigS2.zip › SuppFigure2g.tif]

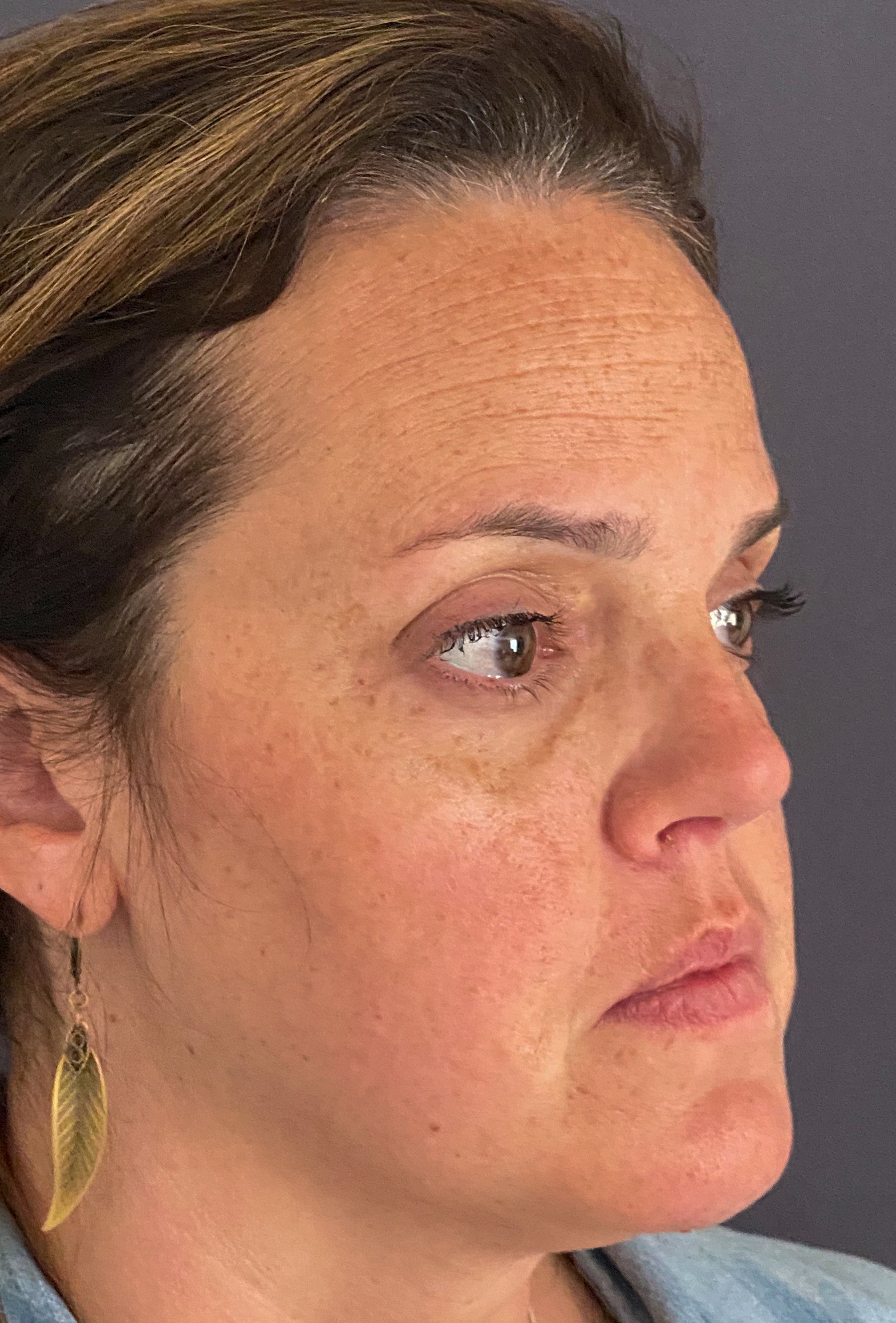

Supplement: Supplemental data [file Suppl_FigS2.zip › SuppFigure2h.tif]

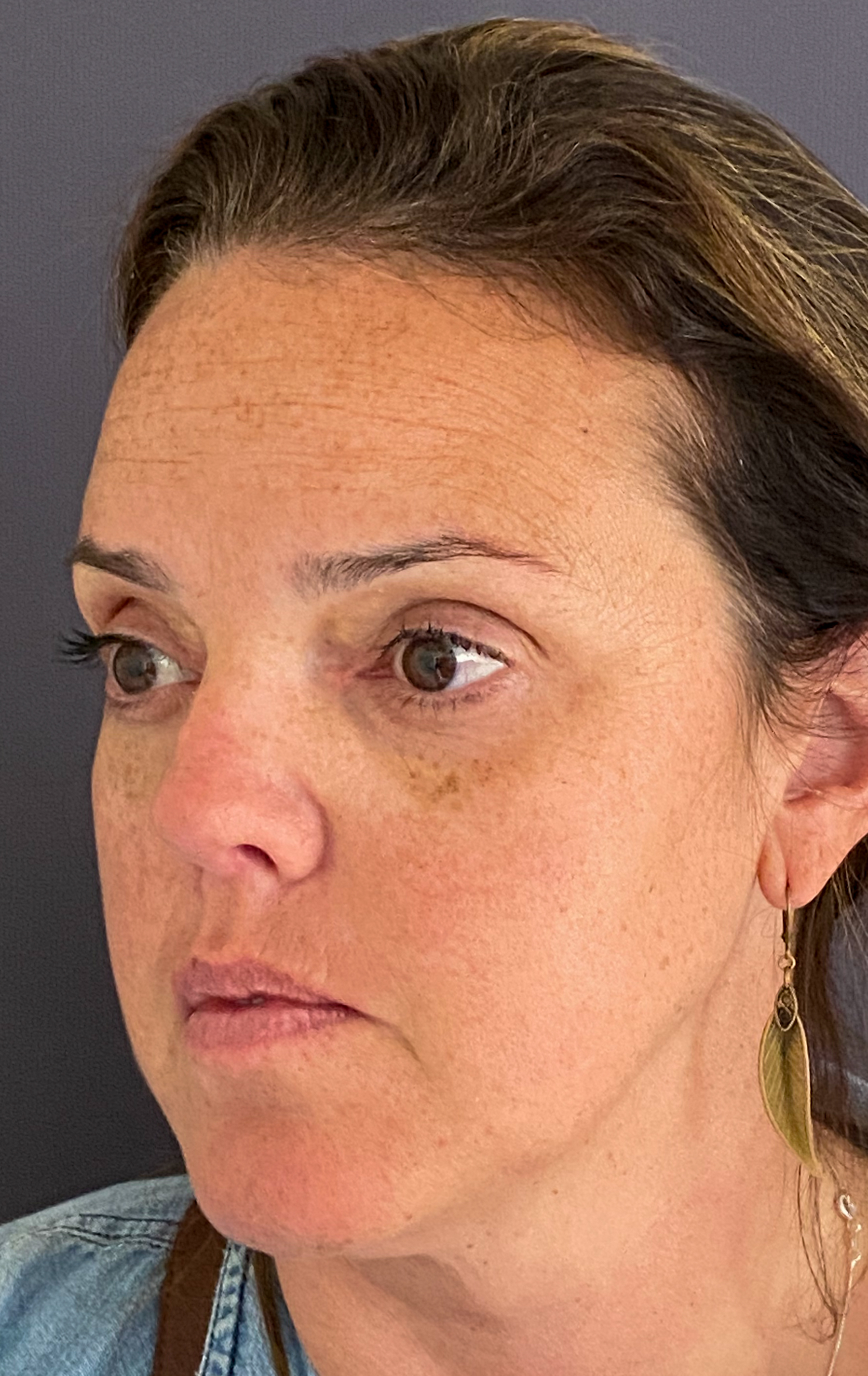

Supplement: Supplemental data [file Suppl_FigS2.zip › SuppFigure2i.tif]

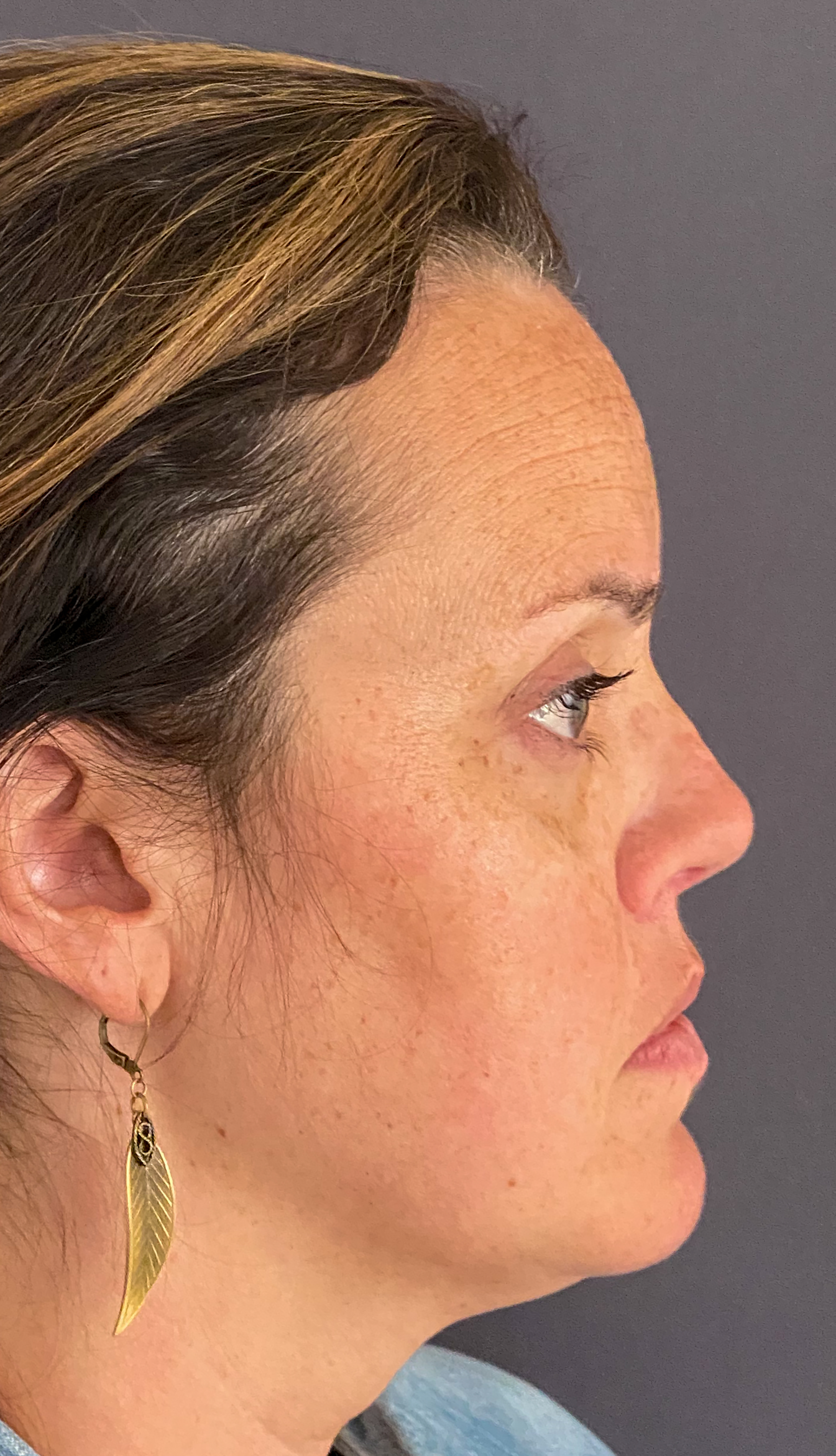

Supplement: Supplemental data [file Suppl_FigS2.zip › SuppFigure2j.tif]

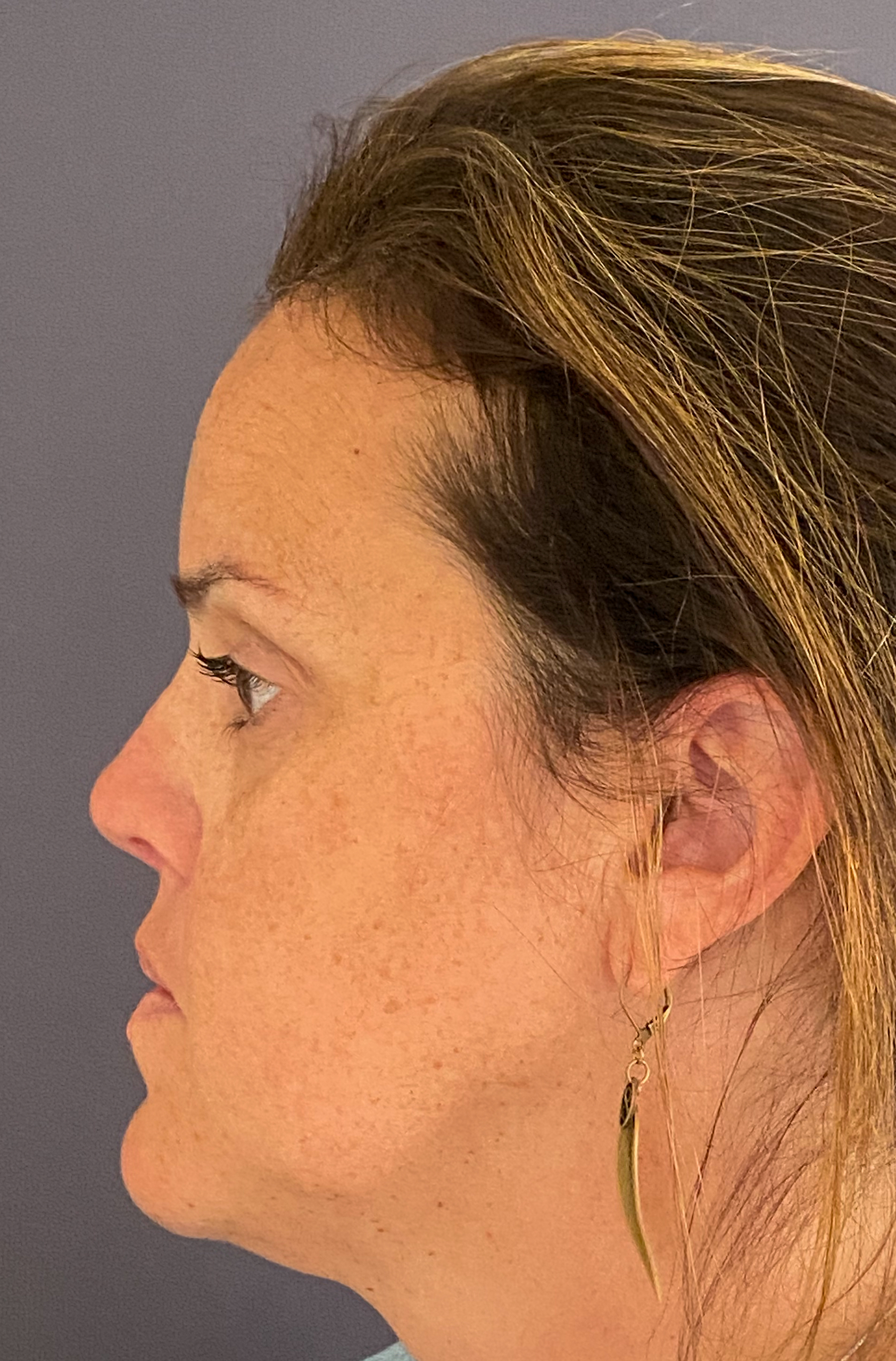

Supplement: Supplemental data [file Suppl_FigS2.zip › SuppFigure2k.tif]

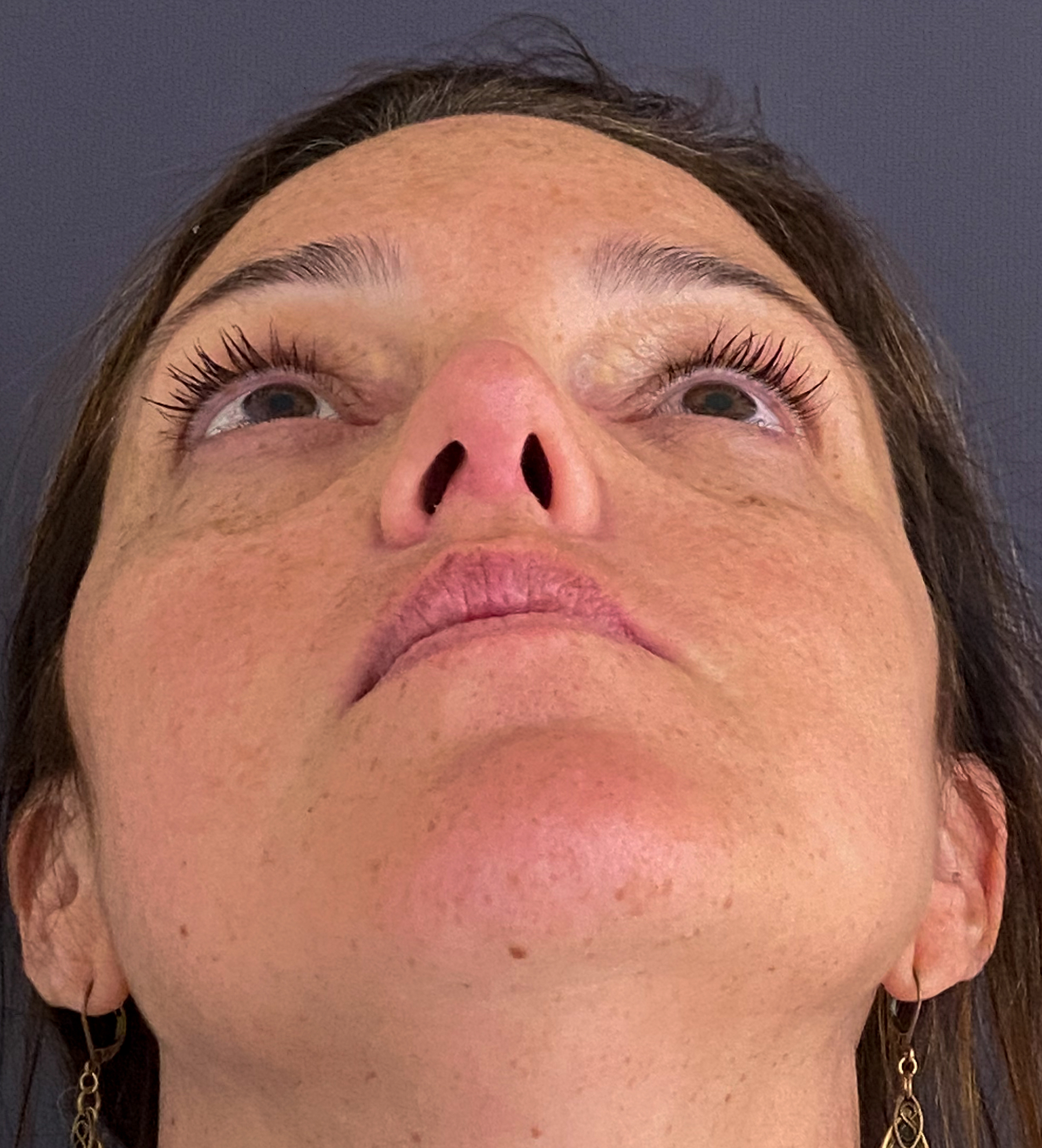

Supplement: Supplemental data [file Suppl_FigS2.zip › SuppFigure2L.tif]

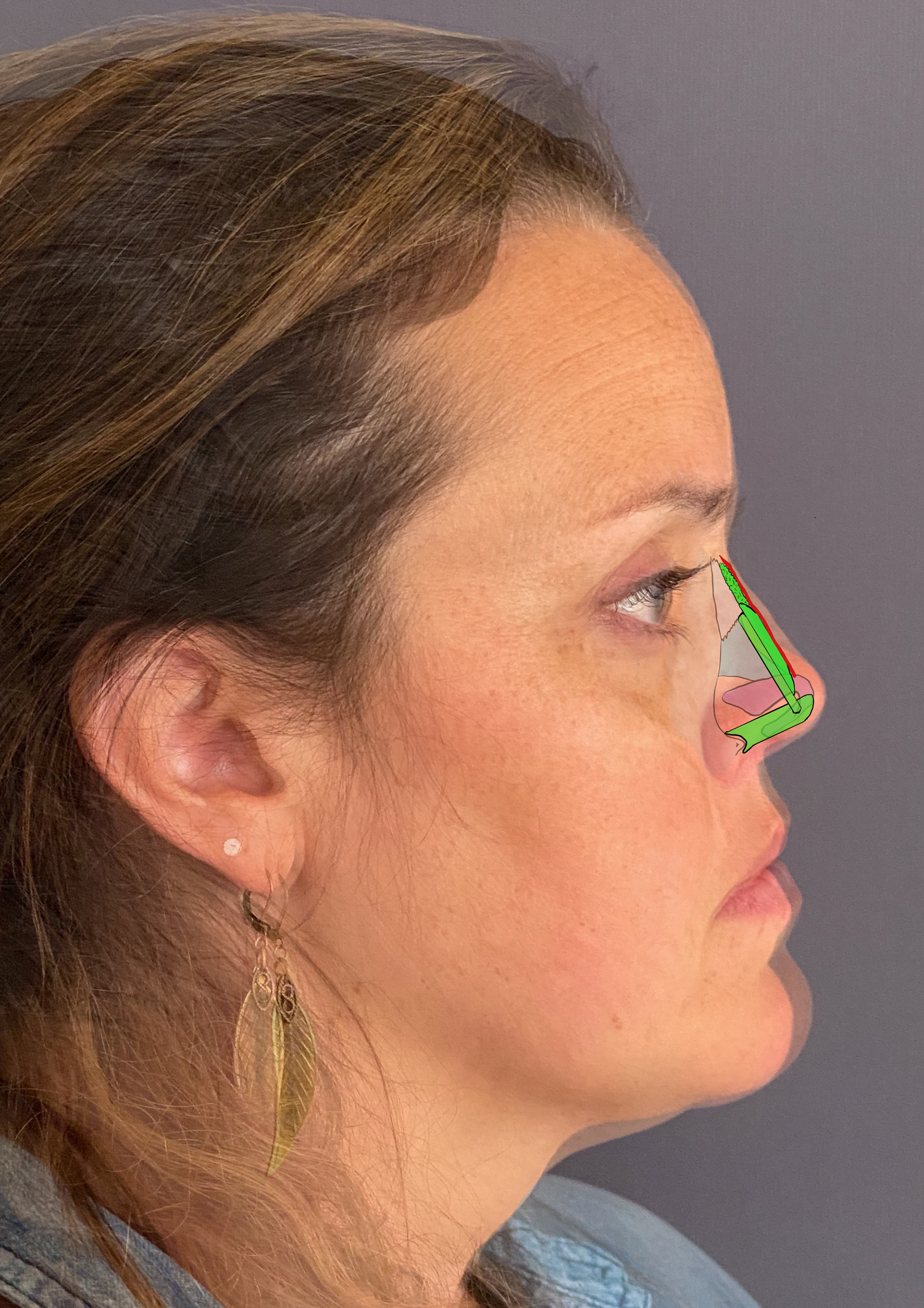

Supplement: Supplemental data [file Suppl_FigS2.zip › SuppFigure2m.tif]

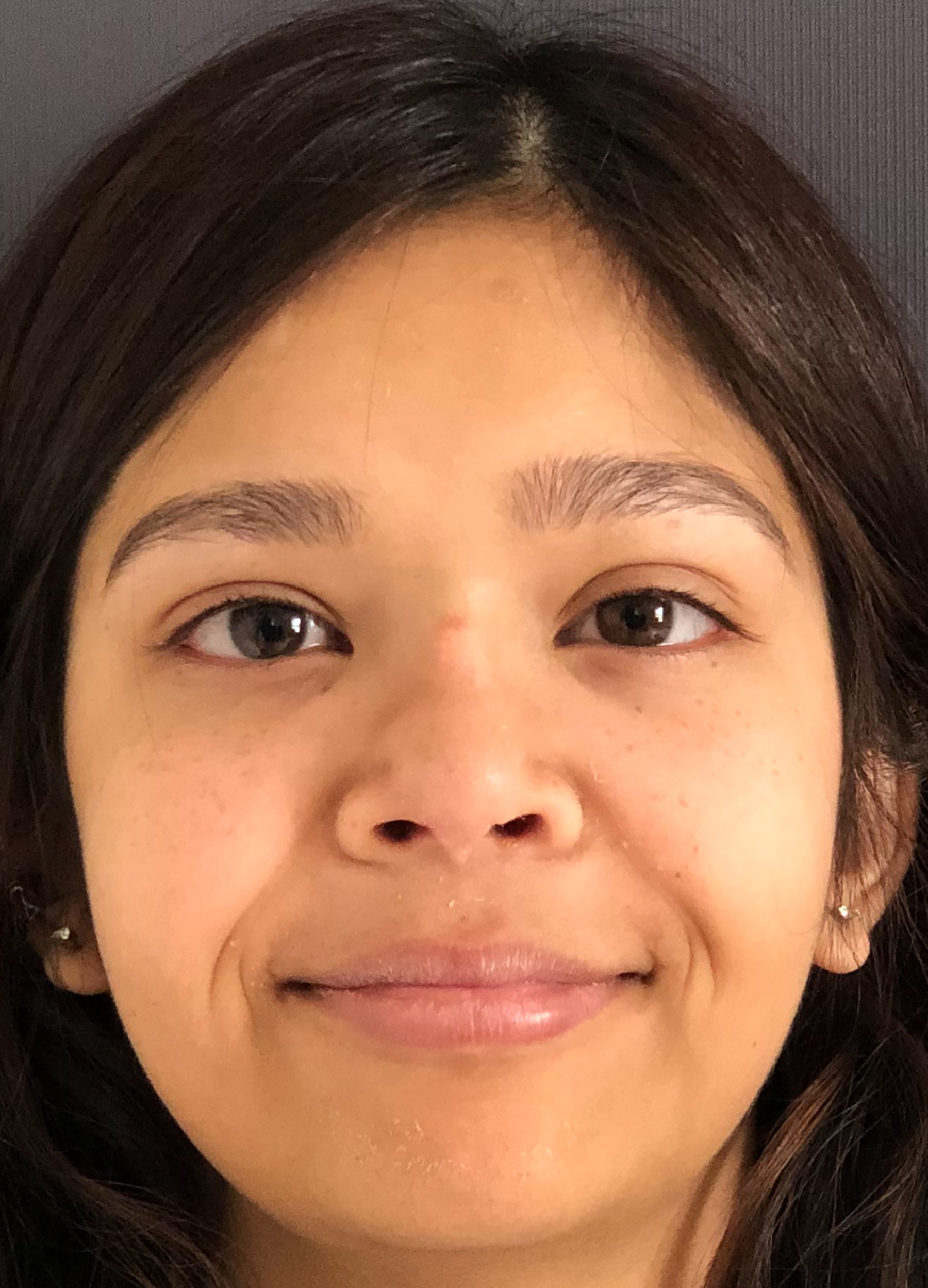

Supplement: Supplemental data [file Suppl_FigS3.zip › SuppFigure3g.tif]

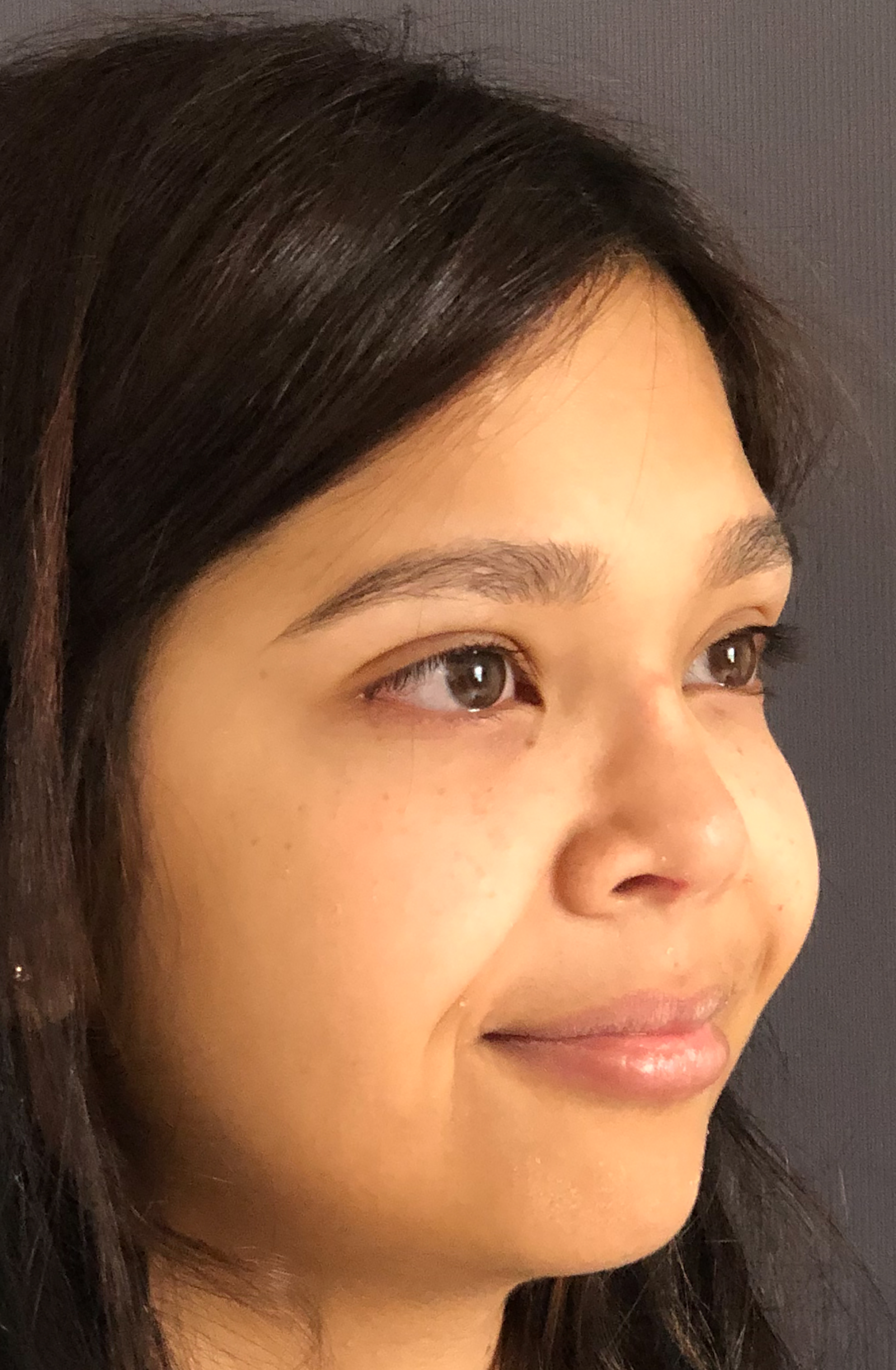

Supplement: Supplemental data [file Suppl_FigS3.zip › SuppFigure3h.tif]

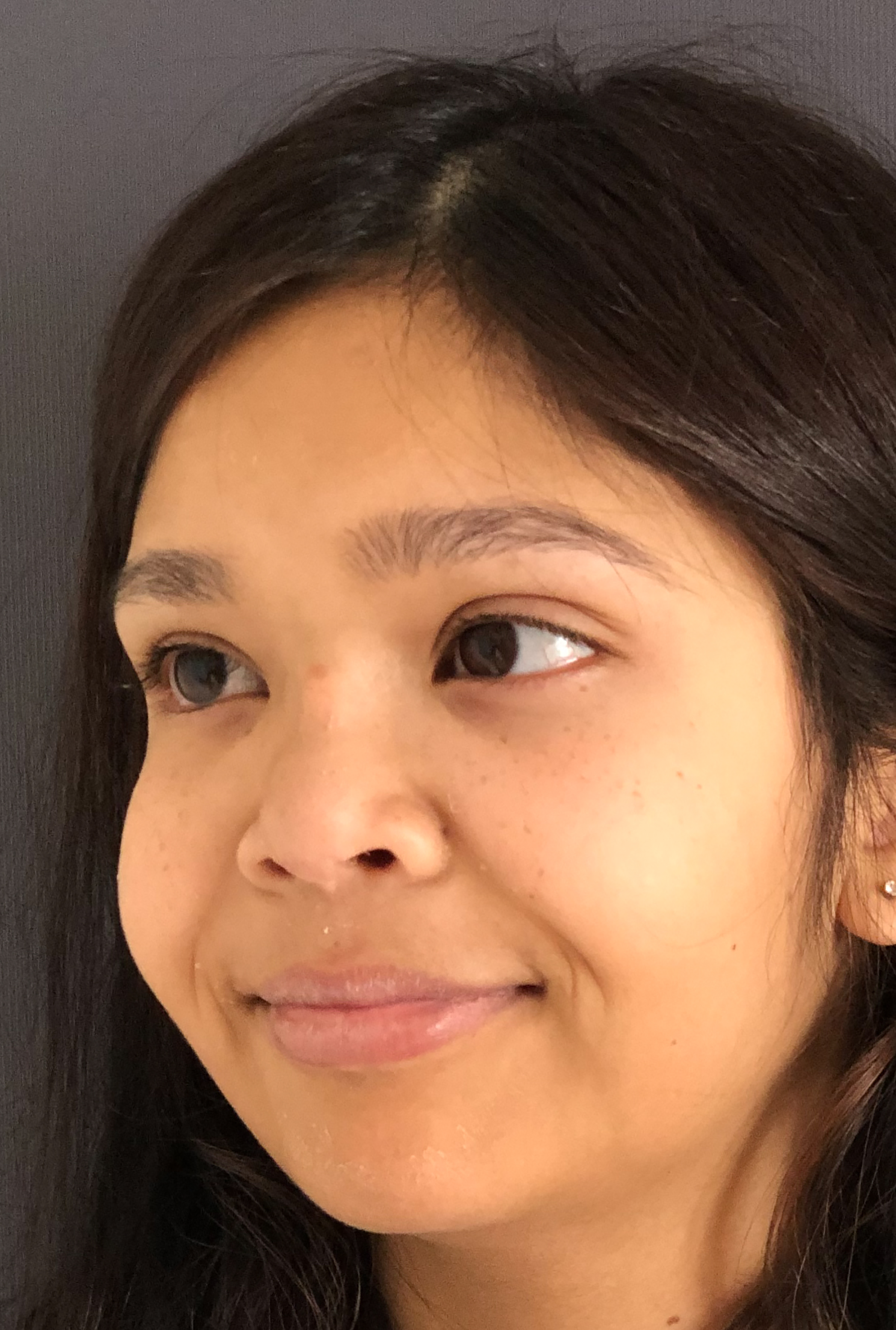

Supplement: Supplemental data [file Suppl_FigS3.zip › SuppFigure3i.tif]

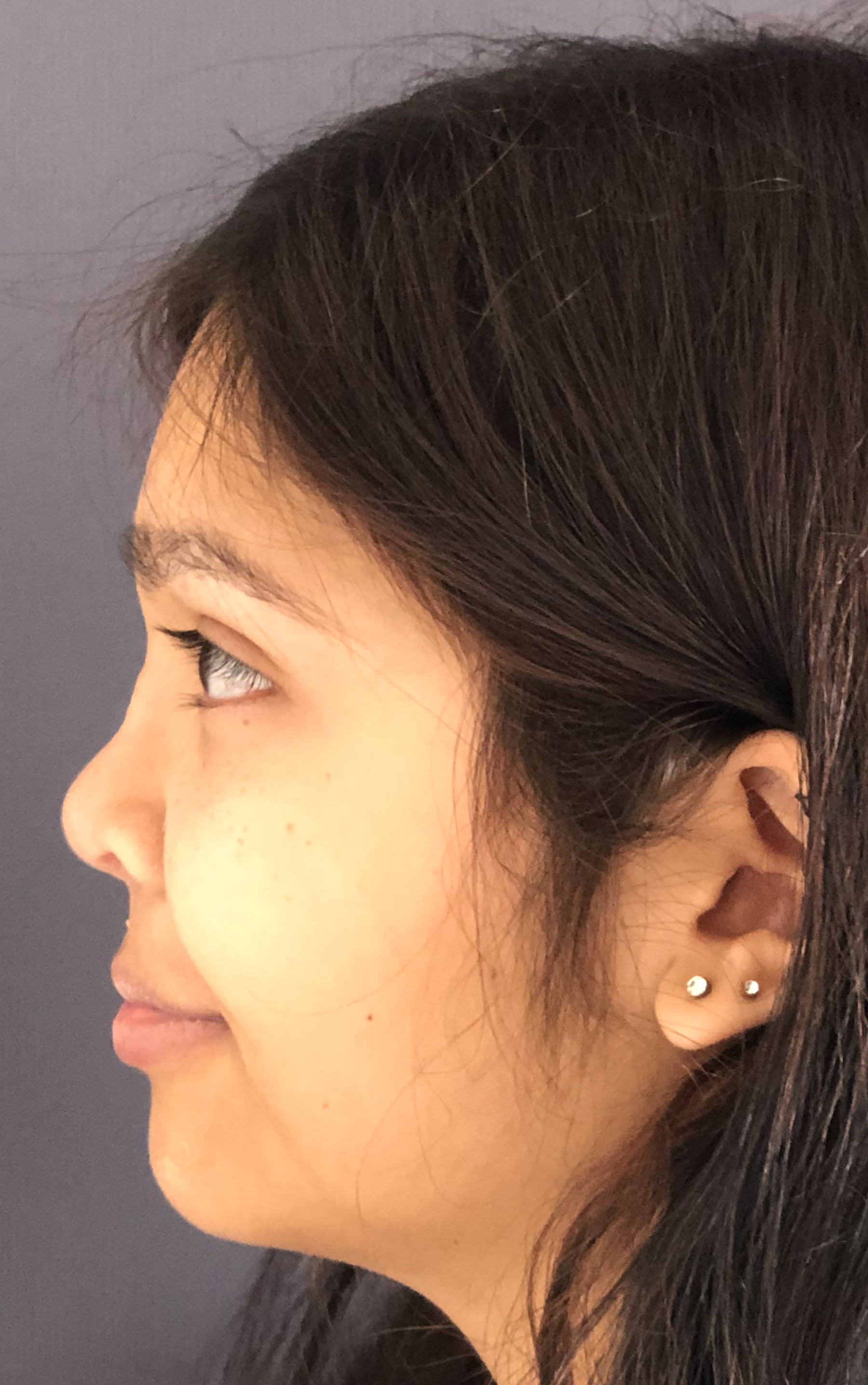

Supplement: Supplemental data [file Suppl_FigS3.zip › SuppFigure3j.tif]

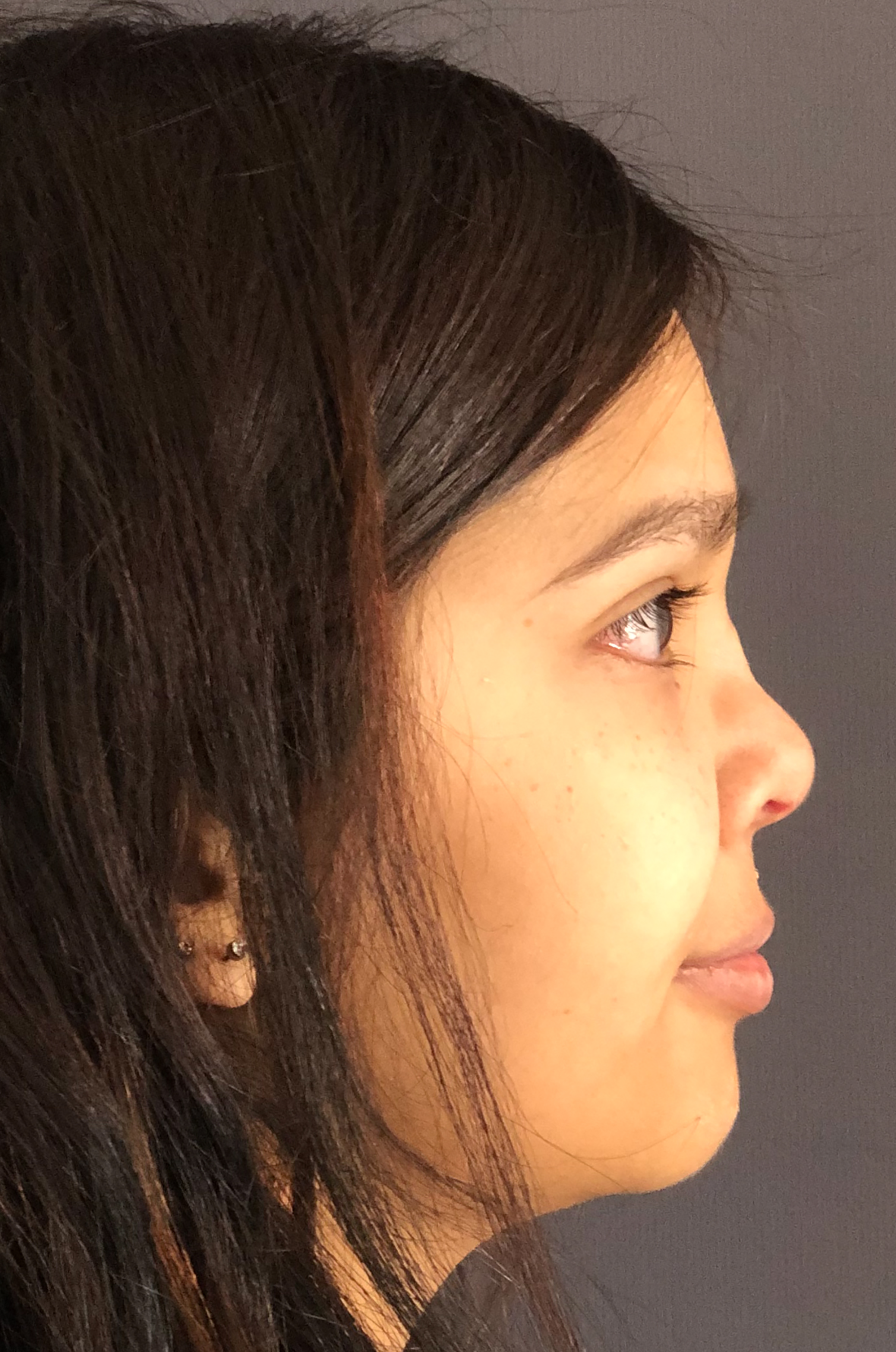

Supplement: Supplemental data [file Suppl_FigS3.zip › SuppFigure3k.tif]

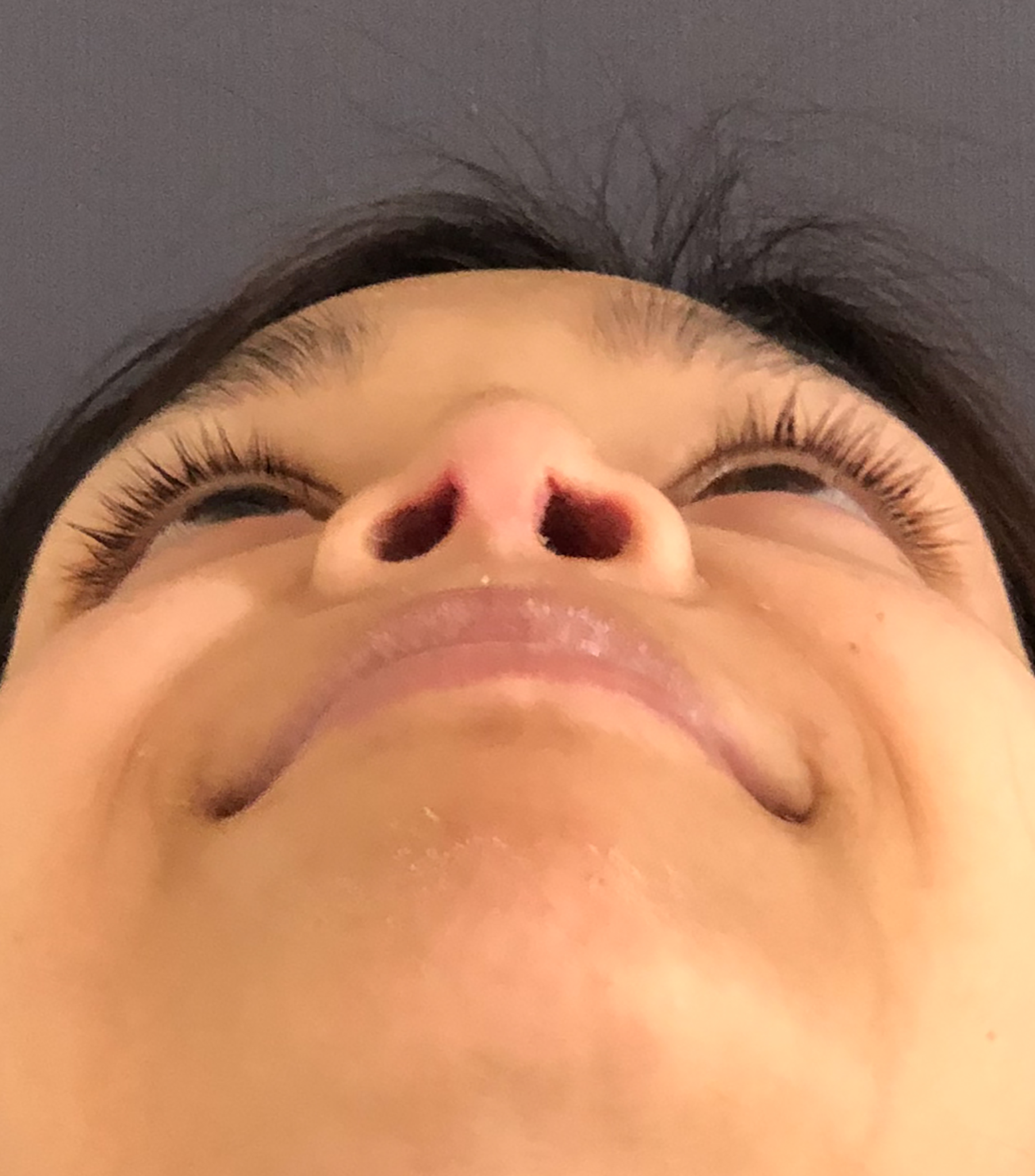

Supplement: Supplemental data [file Suppl_FigS3.zip › SuppFigure3L.tif]

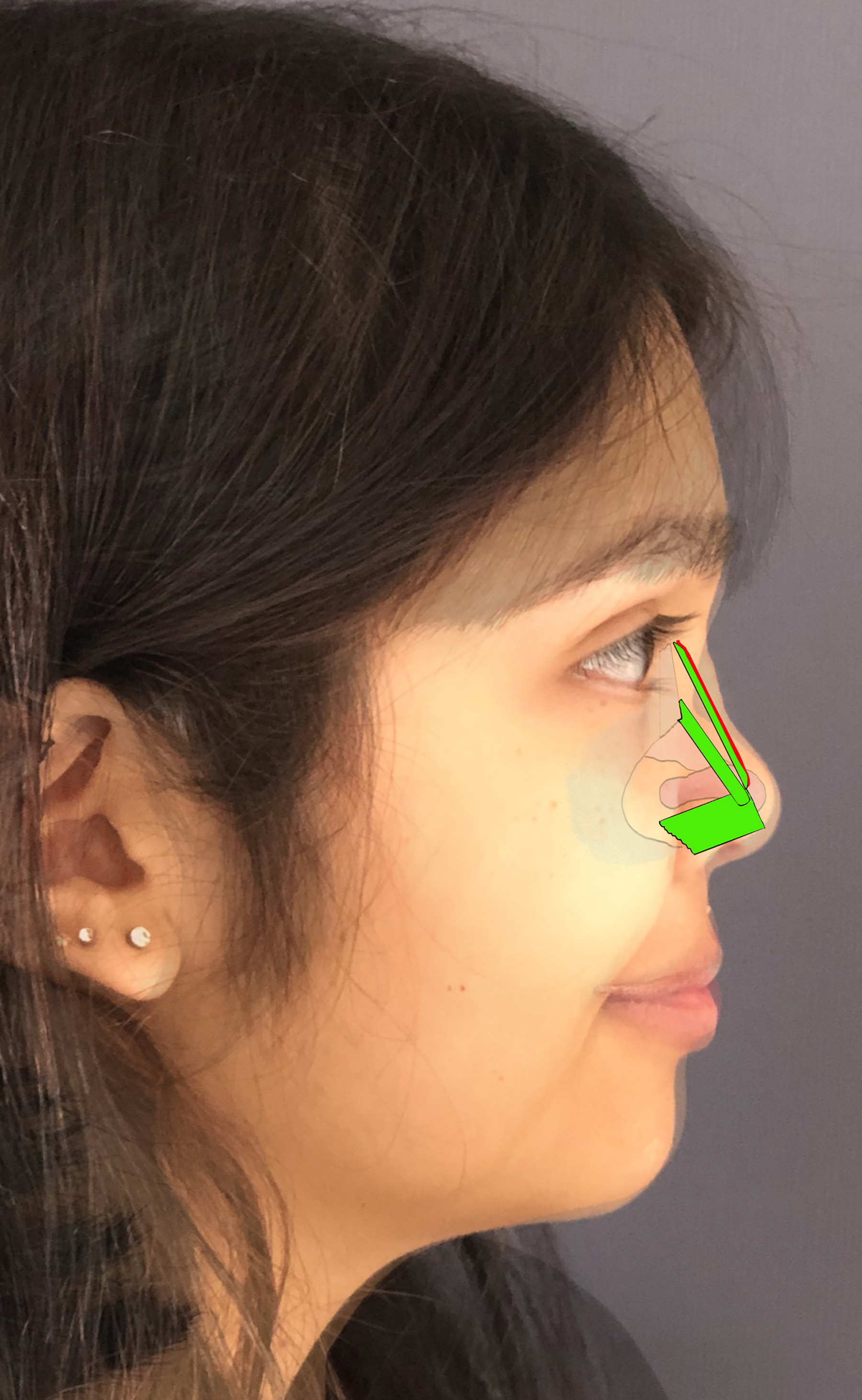

Supplement: Supplemental data [file Suppl_FigS3.zip › SuppFigure3m.tif]

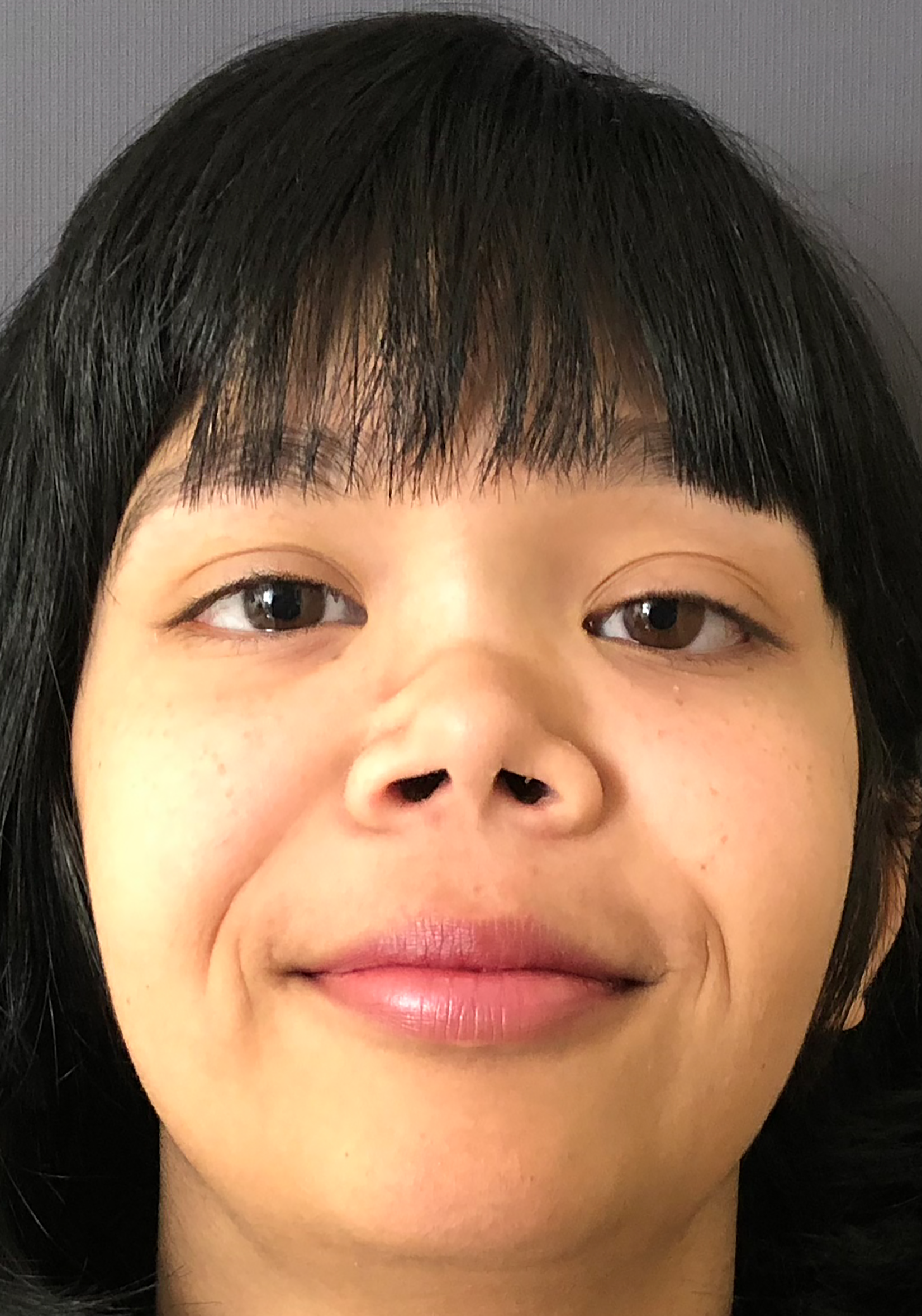

Supplement: Supplemental data [file Suppl_FigS3.zip › SuppFigure3a.tif]

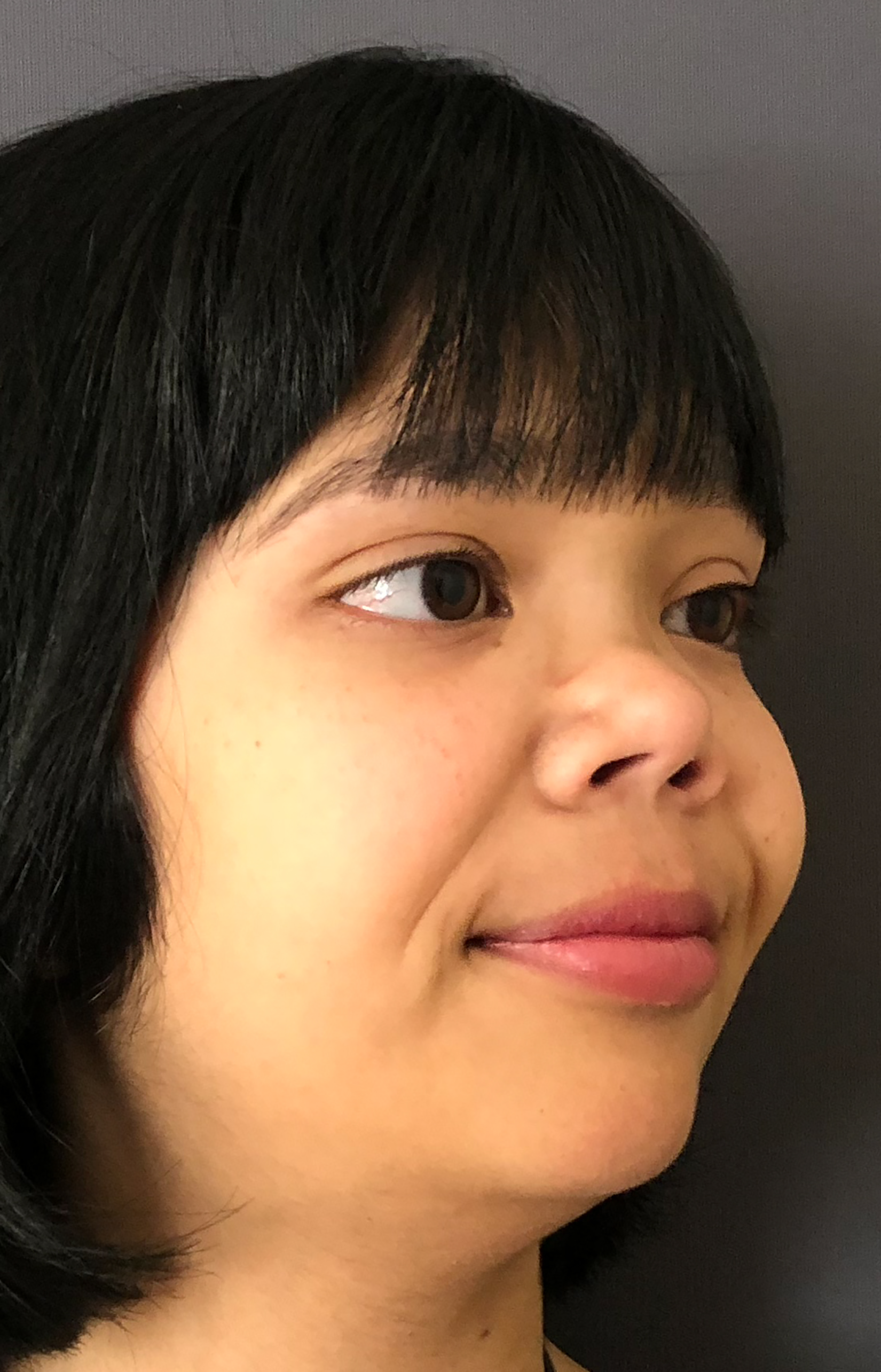

Supplement: Supplemental data [file Suppl_FigS3.zip › SuppFigure3b.tif]

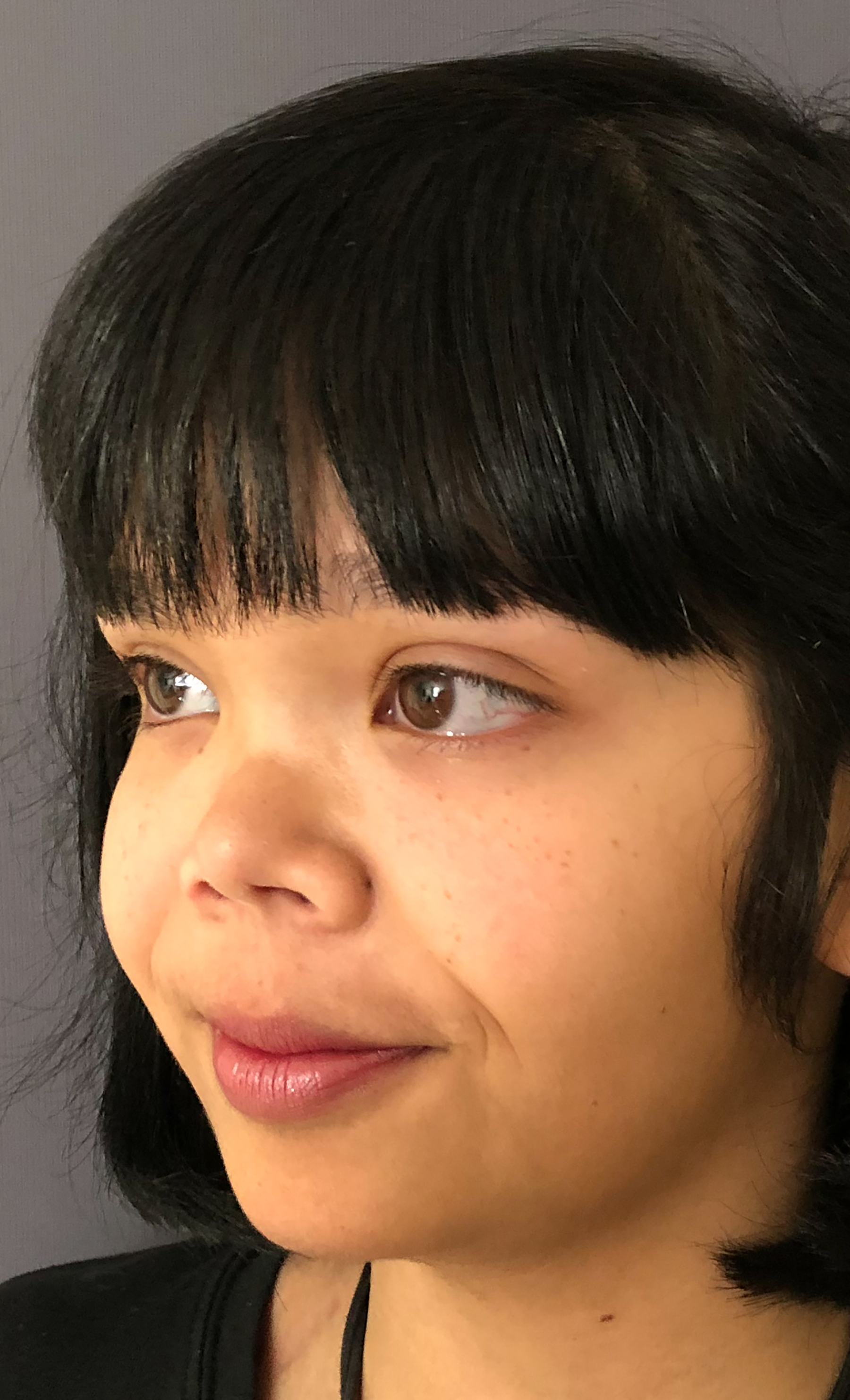

Supplement: Supplemental data [file Suppl_FigS3.zip › SuppFigure3c.tif]

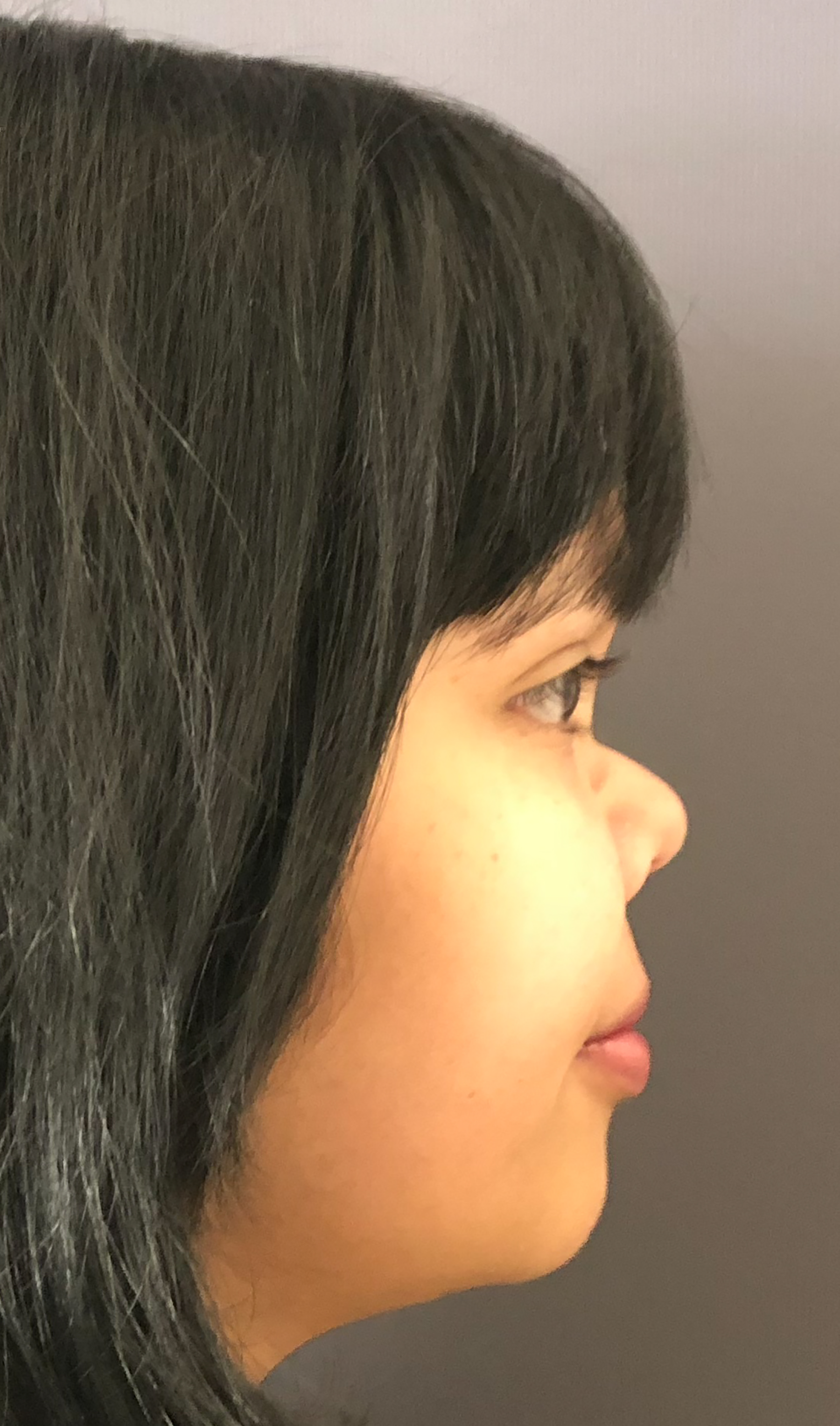

Supplement: Supplemental data [file Suppl_FigS3.zip › SuppFigure3d.tif]

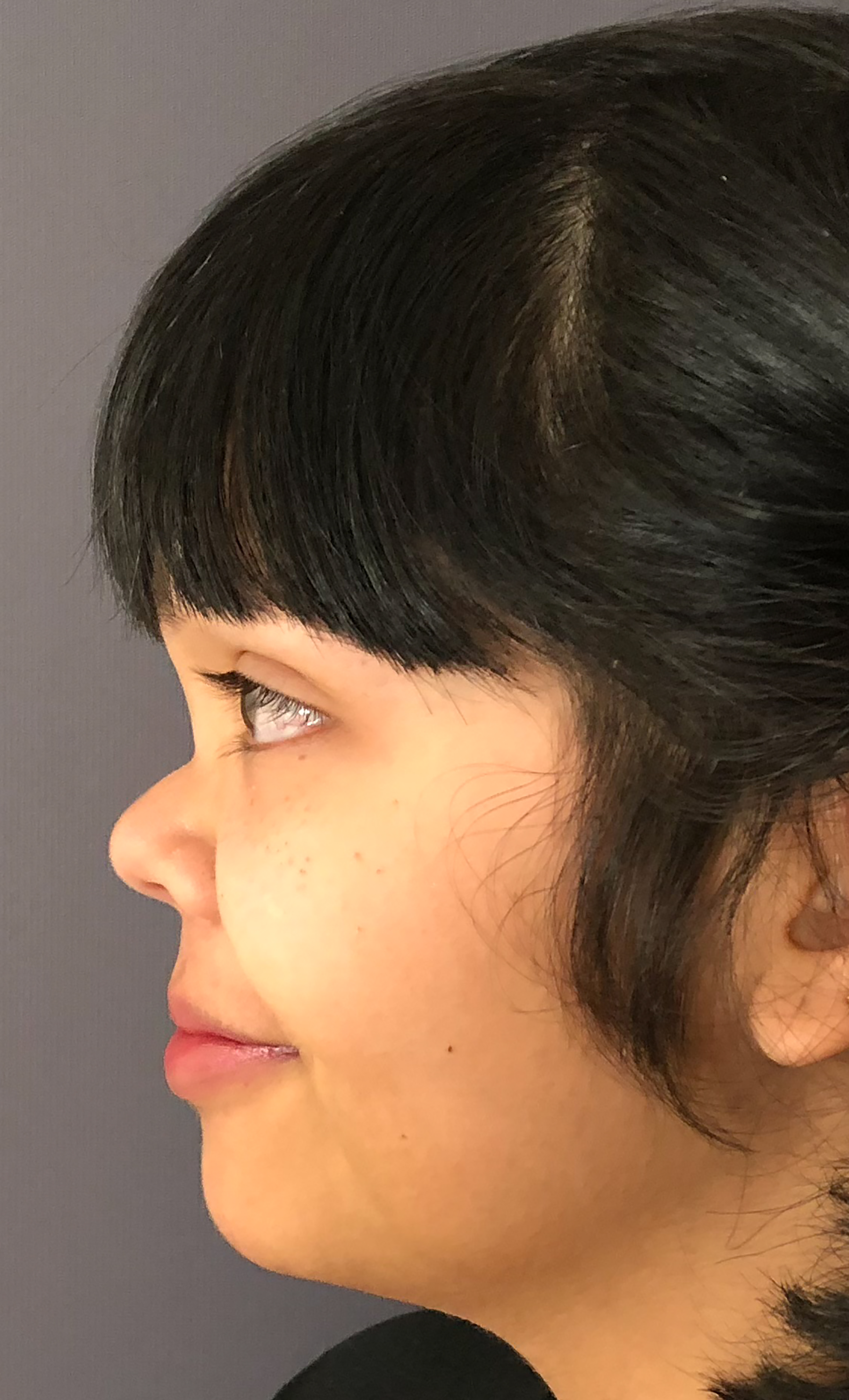

Supplement: Supplemental data [file Suppl_FigS3.zip › SuppFigure3e.tif]

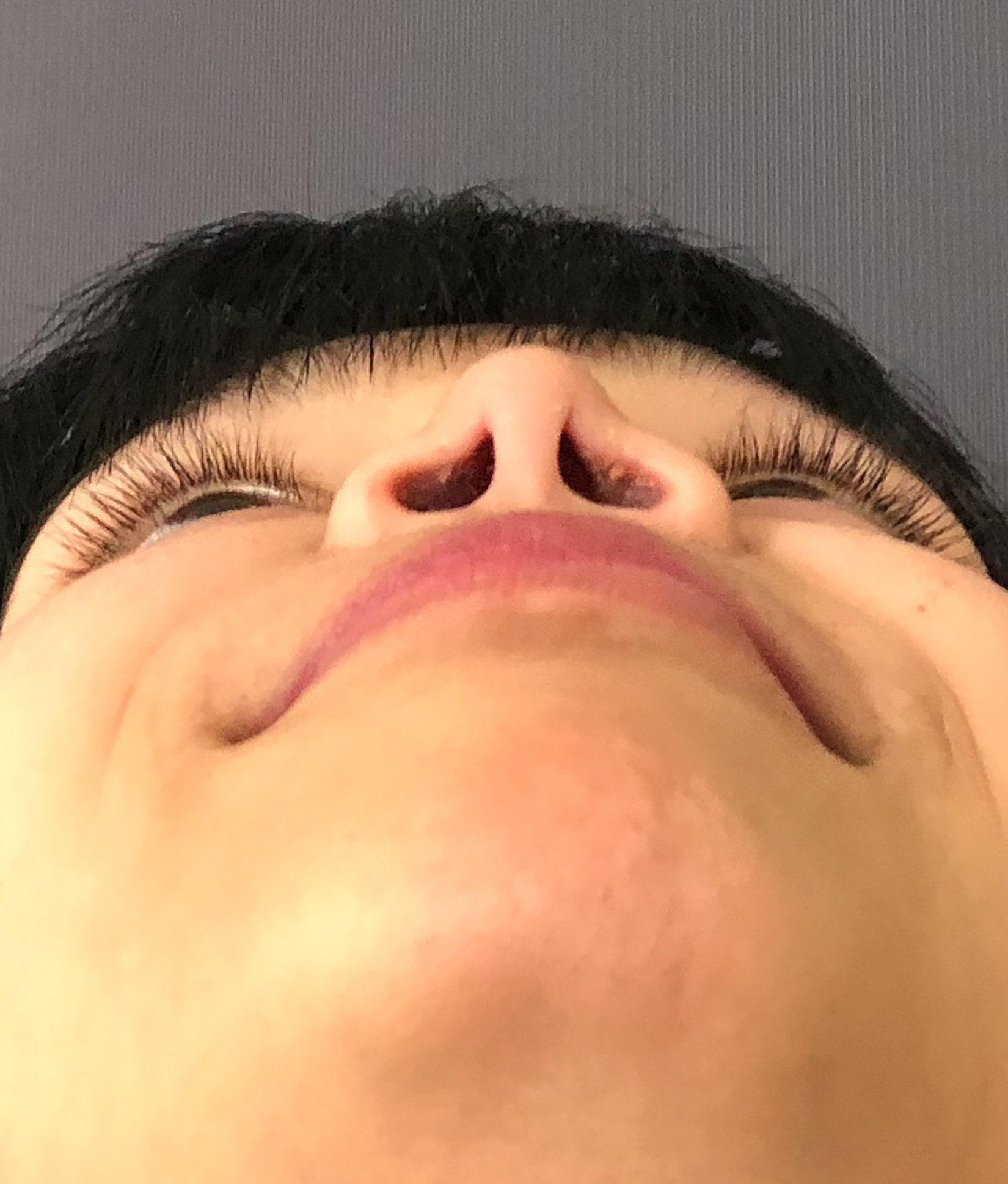

Supplement: Supplemental data [file Suppl_FigS3.zip › SuppFigure3f.tif]

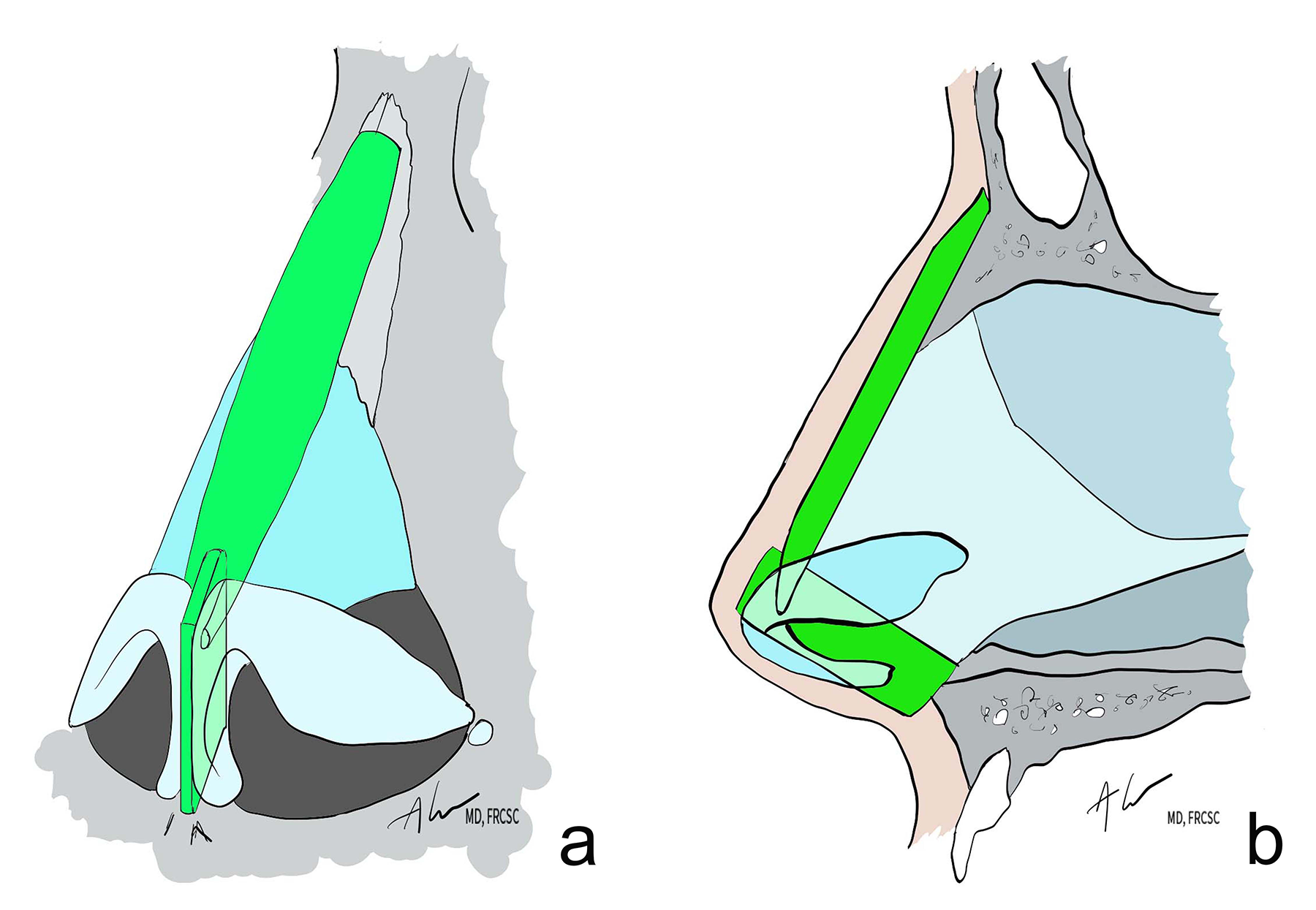


Table 2. Cosmetic satisfaction after 12 postoperative months

8.3%

Supplement: Supplemental data [file Suppl_FigS4.docx]
