# Supplementary material for: Nasal Reconstruction in Granulomatosis with Polyangiitis: A Two Decade Review
Source: Facial Plast Surg Aesthet Med. 2023 Jan 18;25(1):61–7. doi: 10.1089/fpsam.2021.0348 (PMC9885542; doi:10.1089/fpsam.2021.0348)
Supplement: Supplemental data [file Suppl_FigS5.docx]

Table 3. Breathing quality after 12 postoperative months
